# Enanti­opure (*S*)-butan-2-yl *N*-(4-*x*-phen­yl)thio­carbamates, *x* = NO_2_, OCH_3_, F, and Cl

**DOI:** 10.1107/S2056989023002591

**Published:** 2023-03-23

**Authors:** Werner Kaminsky, Max Kaganyuk

**Affiliations:** a Department of Chemistry Univ. of Washington Seattle, WA 98195, USA; Purdue University, USA

**Keywords:** enanti­opure, (*S*)-2but­yl, thio­carbamate, iso­thio­cyanate, crystal structure

## Abstract

The structures of (*S*)-butan-2-yl *N*-(4-nitro­phen­yl)thio­carbamate, C_11_H_14_N_2_O_3_S, (I), (*S*)-butan-2-yl *N*-(4-meth­oxy­phen­yl)thio­carbamate, C_12_H_17_NO_2_S, (II), (*S*)-butan-2-yl *N*-(4-fluoro­phen­yl)thio­carbamate, C_11_H_14_FNOS, (III), and (*S*)-butan-2-yl *N*-(4-chloro­phen­yl)thio­carbamate, C_11_H_14_ClNOS, (IV), all at 100 K, have monoclinic (*P*2_1_) symmetry with two independent mol­ecules in the asymmetric unit. The Flack absolute structure parameters in all cases confirm the absence of inversion symmetry. The structures display N—H⋯S hydrogen bonds, resulting in *R*_2_^2^(8) hydrogen-bonded ring synthons connecting the two independent mol­ecules. Despite the ring synthon, the packing follows two distinct patterns, with (I) and (IV) ‘pancaking’ along the *b-*axis direction, while the other two ‘sandwich’ in layers perpendicular to the *b* axis. Crystal morphologies were determined theoretically *via* the BFDH (Bravais, Friedel, Donnay–Harker) model and agree qualitatively with the experimentally indexed results. One of the butyl substituent of (II) exhibits structural disorder.

## Abstract 2

Enanti­opure (*S*)-butan-2-yl-*N*-(4-*x*-phen­yl)thio­carbamates, *x* = NO_2_, OCH_3_, F, and Cl were synthesized from reacting aryl iso­thio­cyanate with (*S*)-2-butanol to form new chiral crystals as the basis for future research into their non-linear physical and potentially biological properties.

## 1. Chemical context

This research is part of an undergraduate study into creating new chiral model compounds from reacting a chiral moiety with another mol­ecule to combine specific features of both. Initially, iso­thio­cyanates were reacted with α-methyl­benzyl­amine to form chiral thio­urea derivatives (Kaminsky *et al.*, 2010 ▸), whereas here, the poisonous iso­thio­cyanates were reacted with (*S*)-2-butanol to form thio­carbamates with possible protein-docking capability (Bull & Breese, 1978 ▸; Du *et al.* 2020 ▸). Specifically, (*S*)-butan-2-yl-*N*-(4-*x*-phen­yl)thio­carb­amates were synthesized from enanti­opure (*S*)-2-butanol and 4-*x*-phenyl­iso­thio­cyanate, *x* = NO_2_, OCH_3_, F, and Cl. Similar thio­carbamates have been investigated previously for their biological activities (Ghosh & Brindisi, 2015 ▸). 
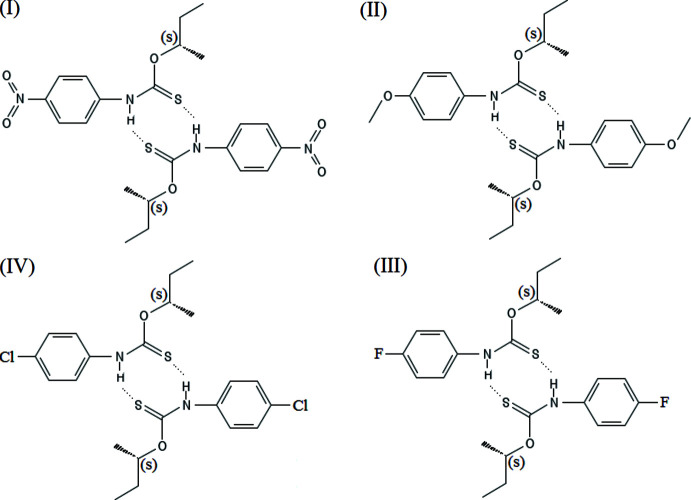


## 2. Structural commentary

Iso­thio­cyanates were selected because of the ease with which the –N=C=S functional group can be reacted with amines or alcohols to form thio­ureas or thio­carbamates, which in turn are well suited for simple crystal-growth studies. The –*R*=S linkage builds out selected hydrogen bonds, structuring the packing of the mol­ecule and thereby enhancing crystal growth. In addition, the sulfur atom has sufficient anomalous scattering capability with Mo radiation, which permits absolute structure determinations *via* single crystal X-ray diffraction. Further, from comparing a series of crystals with small chemical variations, we hoped to gain insight into the functionality of those inter­changed moieties, here NO_2_, OCH_3_, F, and Cl in the 4-*x* location on the structures of the phenyl­thio­carbamates. All four structures crystallize in the chiral space group *P*2_1_ of the monoclinic system. Bond lengths and angles are in the expected ranges. We observed two pairings, where the 4-NO_2_ and 4-Cl crystals exhibited a similarly short *b*-axis, whereas OCH_3_ and F in the 4-*x* location had the longest axis dimensions along *b*. The chirality of the compounds was confirmed by the absolute structure parameters [(I)–(IV): −0.02 (3), −0.04 (4), 0.17 (13), and 0.022 (14), respectively].

## 3. Supra­molecular features

**Figure 1:**
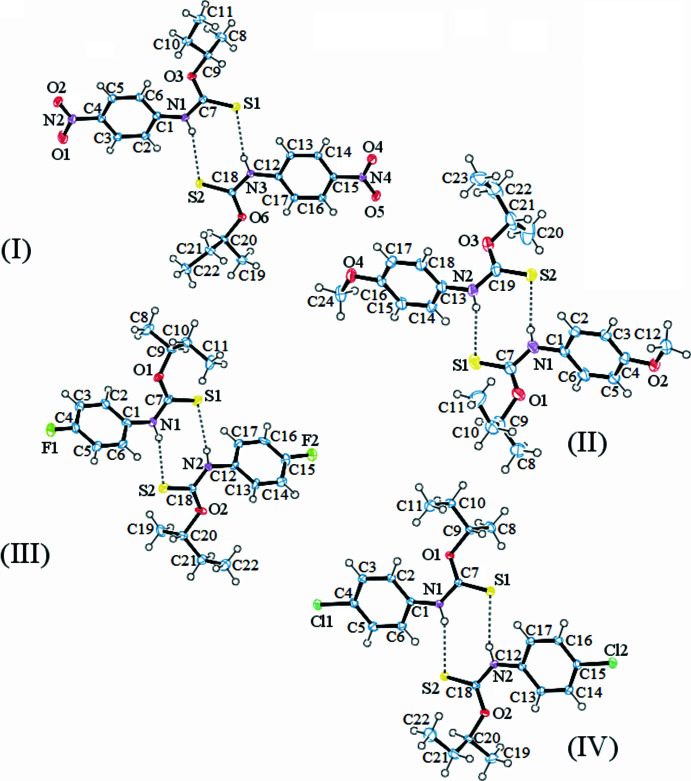
The mol­ecular structure of (I)–(IV), with non-H atoms labeled and 50% probability displacement ellipsoids for non-H atoms. Hydrogen bonds drawn as dashed lines. Disorder omitted for clarity.

In each structure shown in Fig. 1 ▸, pairs of the title mol­ecules organize *via* the thio­amide {⋯H—N—C=S}_2_ into – 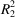 (8) hydrogen-bonded ring synthons (Allen *et al.*, 1999 ▸). All six non-H atoms of the ring synthons are coplanar with r.m.s. deviations from the plane of 0.026 to 0.044 Å between the four synthons. The N⋯S distances of the synthon bonds range from 3.314 (3) to 3.410 (2) Å (Tables 1 ▸–4 ▸ ▸ ▸). Hydrogen-to-acceptor distances are similar as well, however, the *D*—H⋯*A* angles appear to deviate slightly more from a ‘straight’ geometry in compounds (I) and (IV) than in (II) and (III). With the exception of (III), the sulfur atoms act as acceptors for two hydrogen bonds with a major N—H⋯S and a weaker secondary C—H⋯S inter­action, which causes the synthon geometry to shift slightly towards the secondary inter­actions. In (I), we observe a weak inter­action with the *ortho-*C—H of the phenyl groups (C2—H2⋯S2, C13—H13⋯S1). The inter­action is strong enough to also cause the phenyl rings to become coplanar with the synthon plane. In (II), the secondary inter­action is with a proton of the meth­oxy group from a symmetry-related mol­ecule. For (III), consolidating S⋯F non-covalent inter­molecular inter­actions (Thorley & McCulloch, 2018 ▸) are found at 3.62 (1) Å instead of an H⋯S inter­action. The phenyl rings in (IV) are tilted towards the ring synthon plane, causing the *ortho-*C—H distance to the sulfur atoms to be of lesser importance than in (I). Thus, for each case shown, we see a distinctly different bond environment of the sulfur atoms.

**Figure 2:**
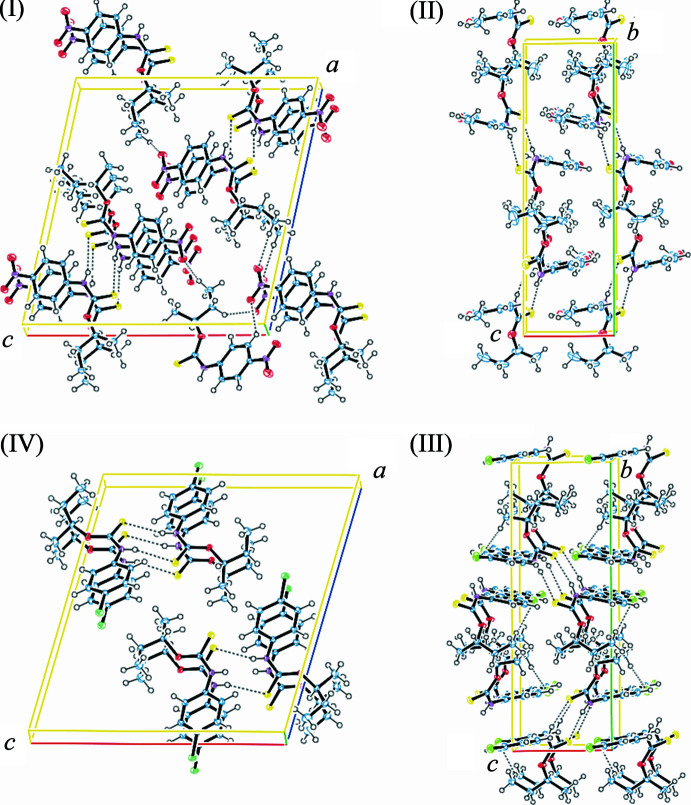
Packing of the structures of this report. (I), (IV): view slightly inclined to the *b* axis·(II), (III): view approximately along the *a* axis. Disorder omitted for clarity.

The packing follows two distinct patterns, with (I) and (IV) ‘pancaking’ along the *b*-axis direction, while the other two ‘sandwich’ in layers perpendicular to the *b*-axis, see Fig. 2 ▸.

**Packing of (II), (III):** The 4-*x*-phenyl moiety is approximately parallel to the *ac* plane. The 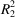 (8) hydrogen-bonded rings orient roughly parallel to the *c* plane in (II) or the *bc* plane in (III). The phenyl­carbamate double layers are separated by layers containing the (*S*)-butan-2-yl moieties. Short distances between the phenyl plane and a symmetry-related OCH_3_ group are seen in (II). As a result of the S—F inter­action in (III), the F atoms are not found as close to a phenyl group, but both are in hydrogen-bonding distance to a methyl group of a symmetry-related butyl moiety.

**Packing of (I), (IV):** The 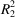 (8) hydrogen-bonded rings are roughly parallel to the *bc* planes. Each dimer stacks entirely like ‘pancakes’ along the short *b*-axis, with a separate stack for the 2_1_ axis-related dimers. The dimers are inclined to **b** so that the NO_2_ group of (I), or Cl of (IV) are found at a short distance to the phenyl of the mol­ecule of the next layer. The NO_2_–phenyl plane distances are not the same for the independent phenyl moieties and are measured at 2.99 (2) and 3.169 (16) Å in (I). In (IV), the Cl–phenyl plane distances are 3.062 (3) and 3.316 (12) Å. These distances are short and may indicate inter­action between the phenyl ring and the 4-*x*-groups, (NO_2_, Cl). One oxygen atom of the NO_2_ in (I) establishes a hydrogen bond with a proton of a symmetry-related phenyl ring.

The different stacking models seem not to correlate with the electronegativity of the ligands, which is generally known to be in decreasing order NO_2_ > F > OCH_3_ > Cl (Pauling, 1932 ▸).

**Figure 3:**
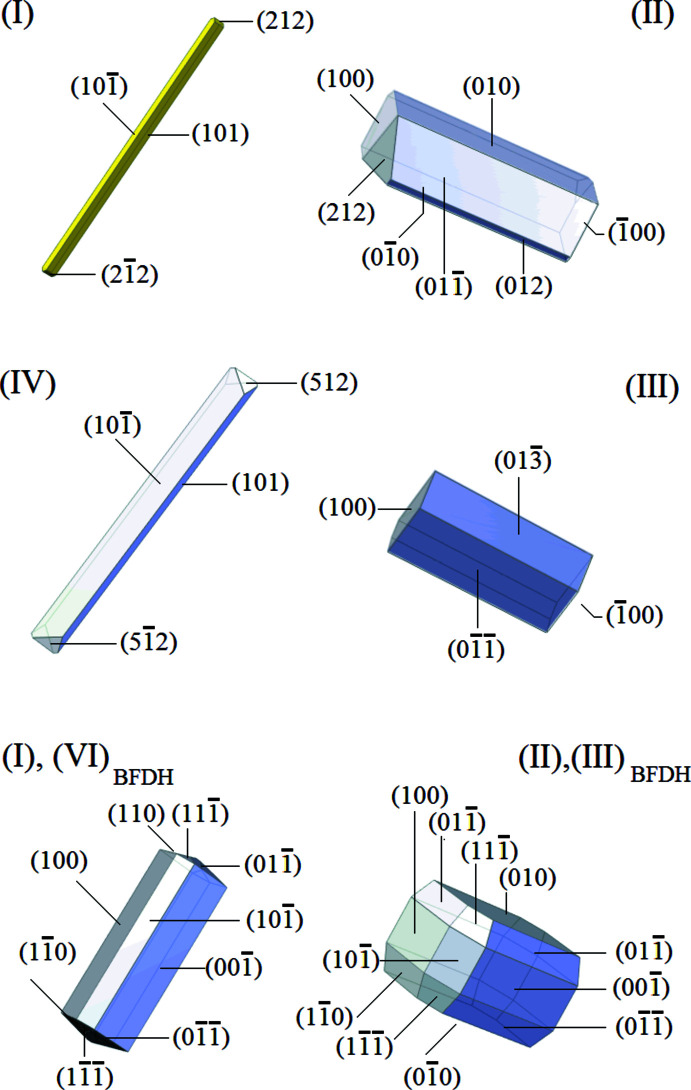
The morphologies of the samples used to obtain structures for this report and the result of BFDH calculations based on the structures.

Morphologies of the four compounds, drawn with *WinXMorph* (Kaminsky, 2005 ▸) are shown in Fig. 3 ▸. For (I), the indexed faces are in decreasing order (increasing central distance): pinacoids 〈1 0 1〉, 〈1 0 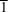 〉, sphenoides 〈2 1 2〉, 〈 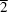 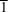 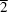 〉. For (IV), the face indexing yielded pinacoids 〈1 0 1〉, 〈1 0 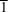 〉, sphenoides 〈5 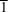 2〉, 〈 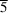 1 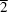 〉. This observation was confirmed qualitatively by BFDH (Bravais, Friedel, Donnay–Harker) model simulations (Bravais, 1866 ▸; Friedel, 1907 ▸; Donnay & Harker, 1937 ▸) using *WinXMorph* (Kaminsky, 2007 ▸) where the dominant crystal facets are pinacoids 〈001〉, 〈100〉, 〈10 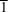 〉, and sphenoides 〈1 1 0〉, 〈0 1 1〉, 〈1 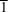 0〉, 〈0 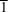 1〉, 〈1 1 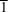 〉, and 〈1 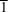 1〉 in decreasing order. For (I) and (IV), it is notable that the 〈0 0 1〉, 〈1 0 0〉 and calculated sphenoids were not observed. For compounds (II) and (III), a more prismatic morphology was observed. The BFDH model yields in both cases, in decreasing face-size order: pinacoids 〈0 1 0〉, 〈1 0 0〉, 〈1 0 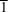 〉, sphenoids 〈1 1 0〉, 〈1 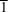 0〉, 〈1 1 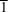 〉, 〈1 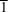 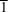 〉. The observed faces in (II) are 〈0 1 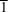 〉, 〈0 1 0〉, 〈0 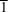 0〉, 〈1 0 0〉, 〈2 1 2〉. Compound (III) grew with 〈0 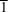 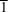 〉, 〈0 1 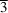 〉, and 〈1 0 0〉 faces.

The BFDH model is entirely based on the metrical and space-group symmetry. It does not account for solvent–surface effects. Thus, differences of growth rates due to such effects in the real samples may often distort the habitus, as well as changing the occurrence of faces.

## 4. Database survey

The structures of this report are not found in the Cambridge Structural Database (CSD version 5.42; Groom *et al.*, 2016 ▸). Earlier, we deposited related structures to the CSD, *viz*. the racemic (*RS*)-butan-2-yl equivalent structures to (I), (II), and (IV), denoted with a prime: (I′): CCDC 2249338, (II’): 2249339, (IV’): 2249336 (Kaganyuk *et al.*, 2023 ▸). Instead of (III′), for which we got only a very low in quality obtained structure, we uploaded the 4-bromo structure (V′), CCDC 2249337. The 4-Cl (IV’) and 4-Br (V′) compounds, both in space group *P*2_1_/*c*, exhibit very similar packing to that of (IV), despite the addition of the glide-plane symmetry. The other two crystallize in the triclinic space group *P* 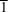 , and only the (*RS*)-butan-2-yl-4-CH_3_ phenyl­thio­carbamate crystal builds out the 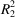 (8) synthon, thus although likely, the thio­carbamates do not always exhibit this feature. A more general search for ‘thio­carbamate’ gave 315 hits, indicating that this substance group has been crystallized moderately often. *Via* a GOOGLE search (March 2023), ‘phenyl­thio­carbamate’ is found 9,370 times. Most of the compounds incorporate a center of symmetry, which is often compatible with an 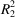 (8) synthon. In fact, the inter­net delivers over 43,000 results when searching for ‘N—H⋯S R22(8) synthon’ (GOOGLE search, March 2023). The number drops considerably, to 93, in a search for ’ring synthon in phenyl­thio­carbamates’. ‘Ring synthon in chiral phenyl­thio­carbamates’ yields only one reasonable result, already included here (Kaminsky *et al.*, 2010 ▸).

## 5. Synthesis and crystallization

All chemicals were obtained from Sigma Aldrich. Compounds (I), (III), and (IV): 4 ml vials were charged with a stir bar, the aryl iso­thio­cyanate [0.100 g, 0.555 mmol (I), 0.653 mmol (III), 0.590 mmol (IV)] and 2(*S*)-butanol (82.3 mg, 1.1 mmol). Using a hot oil bath, the reaction was run at 381 K for 24 h. Compound (II): A 4 mL vial was charged with a stir bar and 2(*S*)-butanol (0.054 g, 0.726 mmol). While stirring, tri­ethyl­amine (0.011 g, 0.109 mmol) was added. After 5 minutes, the aryl iso­thio­cyanate (0.100 g, 0.605 mmol) was added dropwise. The reaction was allowed to continue for 24 h at 358 K. Subsequently, for all four compounds, the vials, after allowing to cool, were covered with filter paper and left in a vacuum oven at 343 K. The crude product was purified by flash column chromatography, and eluted with 1:4 ethyl acetate/hexane. Fractions were collected in 13 × 100 mm test tubes and were spotted for thin layer chromatography to locate the product. The fractions containing the product were rotovaped in a 25 ml round-bottom flask. The solid found in low yields was redissolved in a 1:4 methanol/ethanol solution and crystals grew *via* slow evaporation. (I): ^1^H NMR (300 MHz, CDCl_3_): δ 9.2638 (*bs*, 1H), 8.1926 (*d*, *J* = 7.1 Hz, 2H), 7.5520 (*bs*, 2H), 5.5528 (*m*, 1H), 1.7044 (*m*, 2H), 1.4038 (*d*, *J* = 6.3 Hz, 3H), 0.9634 (*t*, *J* = 7.4 Hz, 3H). (II): ^1^H NMR (300 MHz,(CD_3_)_2_CO): δ 9.7438 (*s*, 1H), 7.5813 (*m*, 2H), 6.9070 (*d*, *J* = 9.1 Hz, 2H), 5.4819 (*bs*, 3H), 3.7847 (*s*, 3H), 1.7022 (*m*, 2H), 1.2948 (*s*, 3H), 0.9184 (*t*, *J* = 7.4, 3H). (III): ^1^H NMR (300 MHz, CDCl3): δ 8.8978 (*bs*, 1H), 7.2240 (*bs*, 2H), 7.0147 (*t*, *J* = 8.5 Hz, 2H), 5.0768 (*m*, 1H), 1.7280 (*m*, 2H), 1.3461 (*d*, *J* = 6.5 Hz, 3H), 0.9316 (*t*, *J* = 7.5 Hz, 3H). (IV): ^1^H NMR (300 MHz, CDCl_3_): δ 8.7150 (*bs*, 1H), 7.2918 (*d*, *J* = 8.6 Hz, 2H), 7.2130 (*bs*, 2H), 5.5199 (*m*, 1H), 1.7269 (*m*, 2H), 1.3640 (*d*, *J* = 6.2 Hz, 3H), 0.9472 (*t*, *J* = 7.4 Hz, 3H).

## 6. Refinement

Crystal data, data collection, and structure refinement details are summarized in Table 5 ▸. Hydrogen atoms on carbon atoms were positioned geometrically, using a riding model, with C—H = 0.95–1.00Å. *U*_iso_(H) = 1.2 (1.5 for methyl groups) times *U*_eq_(C). The nitro­gen protons were refined positionally, with *U*_iso_(H) = 1.2*U*_eq_(N). The two phenyl groups of the independent mol­ecules of (III) were optimized to enhance the C—C bond precision with the C—C distance at 1.39 Å (AFIX 66). In (II), one of the two (*S*)-butan-2-yl moieties appeared threefold disordered, requiring restraint of the displacement parameters with a SIMU 0.01 command. One atom (C8) was constrained to the same displacement parameter for each fraction with EADP. The disordered geometries were linked through a SAME command to the geometry of the ordered moiety of the other mol­ecule, and distances of O1 to C8, C8*B* and C8*C* were restrained to be similar (SADI), all with default esds. The occupancies of the three fractions were 0.444 (4), 0.354 (4), and 0.202 (4).

## Supplementary Material

Crystal structure: contains datablock(s) I, II, III, IV, New_Global_Publ_Block. DOI: 10.1107/S2056989023002591/zl5044sup1.cif

Structure factors: contains datablock(s) I. DOI: 10.1107/S2056989023002591/zl5044Isup5.hkl

Structure factors: contains datablock(s) II. DOI: 10.1107/S2056989023002591/zl5044IIsup6.hkl

Structure factors: contains datablock(s) III. DOI: 10.1107/S2056989023002591/zl5044IIIsup7.hkl

Structure factors: contains datablock(s) IV. DOI: 10.1107/S2056989023002591/zl5044IVsup8.hkl

Supporting information file. DOI: 10.1107/S2056989023002591/zl5044Isup6.cml

Supporting information file. DOI: 10.1107/S2056989023002591/zl5044IIsup7.cml

Supporting information file. DOI: 10.1107/S2056989023002591/zl5044IIIsup8.cml

Supporting information file. DOI: 10.1107/S2056989023002591/zl5044IVsup9.cml

CCDC references: 2249641, 2249640, 2249639, 2249638

Additional supporting information:  crystallographic information; 3D view; checkCIF report

## Figures and Tables

**Table 1:** Hydrogen-bond geometry (Å, °) for (I)

*D*—H⋯*A*	*D*—H	H⋯*A*	*D*⋯*A*	*D*—H⋯*A*
N1—H1*N*⋯S2	0.80 (2)	2.62 (2)	3.3762 (19)	159 (2)
N3—H3*N*⋯S1	0.85 (2)	2.57 (2)	3.4095 (18)	166 (2)
C2—H2⋯S2	0.95	2.87	3.592 (2)	134
C13—H13⋯S1	0.95	2.81	3.611 (2)	142
C5—H5⋯O2^i^	0.95	2.53	3.203 (3)	128

Symmetry code: (i) 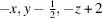 .

**Table 2:** Hydrogen-bond geometry (Å, °) for (II)

*D*—H⋯*A*	*D*—H	H⋯*A*	*D*⋯*A*	*D*—H⋯*A*
N1—H1*N*⋯S2	0.82 (4)	2.53 (4)	3.347 (3)	171 (4)
N2—H2*N*⋯S1	0.86 (4)	2.47 (4)	3.314 (3)	165 (4)
C12—H12*B*⋯S1^i^	0.98	2.86	3.793 (4)	158
C24—H24*B*⋯S2^ii^	0.98	2.86	3.819 (4)	167
C18—H18⋯S2^iii^	0.95	2.98	3.730 (4)	136
C10*B*—H10*C*⋯S1	0.99	2.96	3.445 (11)	112

Symmetry codes: (i) 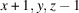 ; (ii) 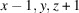 ; (iii) 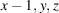 .

**Table 3:** Hydrogen-bond geometry (Å, °) for (III)

*D*—H⋯*A*	*D*—H	H⋯*A*	*D*⋯*A*	*D*—H⋯*A*
N1—H1*N*⋯S2	0.86 (5)	2.51 (5)	3.341 (8)	164 (8)
N2—H2*N*⋯S1	0.86 (5)	2.50 (5)	3.336 (7)	165 (7)
C8—H8*A*⋯F1^i^	0.98	2.59	3.494 (10)	154

Symmetry code: (i) 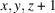 .

**Table 4:** Hydrogen-bond geometry (Å, °) for (IV)

*D*—H⋯*A*	*D*—H	H⋯*A*	*D*⋯*A*	*D*—H⋯*A*
N1—H1⋯S2	0.83 (2)	2.511 (19)	3.3163 (13)	163.0 (18)
N2—H2⋯S1	0.829 (19)	2.563 (19)	3.3645 (13)	162.8 (17)
C6—H6⋯S2	0.95	2.99	3.5961 (17)	123
C17—H17⋯S1	0.95	2.97	3.6122 (16)	127
C2—H2*A*⋯O1	0.95	2.38	2.8539 (18)	111
C13—H13⋯O2	0.95	2.29	2.8197 (19)	114

**Table 5:** Experimental details

	(I)	(II)	(III)	(IV)
Crystal data				
Chemical formula	C_11_H_14_N_2_O_3_S	C_12_H_17_NO_2_S	C_11_H_14_FNOS	C_11_H_14_ClNOS
*M*_r_	254.3	239.32	227.29	243.74
Crystal system, space group	Monoclinic, *P*2_1_	Monoclinic, *P*2_1_	Monoclinic, *P*2_1_	Monoclinic, *P*2_1_
Temperature (K)	100	100	100	100
*a*, *b*, *c* (Å)	16.052 (2), 4.7635 (6), 16.853 (2)	6.6973 (5), 21.2076 (17), 9.1899 (7)	6.9723 (13), 20.166 (3), 8.2818 (14)	15.4173 (15), 5.0170 (5), 16.2502 (15)
β (°)	101.702 (8)	102.868 (5)	99.403 (13)	105.592 (5)
*V* (Å^3^)	1261.9 (3)	1272.49 (17)	1148.8 (3)	1210.7 (2)
*Z*	4	4	4	4
Radiation type	Mo *K*α	Mo *K*α	Mo *K*α	Mo *K*α
μ (mm^−1^)	0.26	0.24	0.27	0.46
Crystal size (mm)	0.6 × 0.12 × 0.06	0.6 × 0.48 × 0.2	0.5 × 0.1 × 0.05	0.6 × 0.12 × 0.11

Data collection				
Diffractometer	Bruker APEXII	Bruker APEXII	Bruker APEXII	Bruker APEXII
Absorption correction	Numerical (*SADABS*; Krause *et al.*, 2015 ▸)	Numerical (*SADABS*; Krause *et al.*, 2015 ▸)	Multi-scan (*SADABS*; Krause *et al.*, 2015 ▸)	Numerical (*SADABS*; Krause *et al.*, 2015 ▸)
*T*_min_, *T*_max_	0.959, 1	0.657, 0.745	0.863, 1	0.954, 1
No. of measured, independent and observed [*I* > 2σ(*I*)] reflections	37641, 9553, 7648	21043, 7694, 5666	10466, 5263, 2448	46534, 9292, 8469
*R*_int_	0.047	0.048	0.171	0.029
(sin θ/λ)_max_ (Å^−1^)	0.772	0.720	0.650	0.772

Refinement				
*R*[*F* ^2^ > 2σ(*F* ^2^)], *wR*(*F* ^2^), *S*	0.039, 0.084, 1.01	0.051, 0.113, 1.01	0.068, 0.143, 0.95	0.027, 0.066, 1.04
No. of reflections	9553	7694	5263	9292
No. of parameters	317	368	257	281
No. of restraints	1	293	2	1
H-atom treatment	H atoms treated by a mixture of independent and constrained refinement	H atoms treated by a mixture of independent and constrained refinement	H atoms treated by a mixture of independent and constrained refinement	H atoms treated by a mixture of independent and constrained refinement
Δρ_max_, Δρ_min_ (e Å^−3^)	0.31, −0.28	0.51, −0.42	0.45, −0.52	0.36, −0.22
Absolute structure	Flack *x* determined using 2891 quotients [(*I* ^+^)−(*I* ^−^)]/[(*I* ^+^)+(*I* ^−^)] (Parsons *et al.*, 2013 ▸)	Flack *x* determined using 2891 quotients [(*I* ^+^)−(*I* ^−^)]/[(*I* ^+^)+(*I* ^−^)] (Parsons *et al.*, 2013 ▸)	Flack *x* determined using 2891 quotients [(*I* ^+^)−(*I* ^−^)]/[(*I* ^+^)+(*I* ^−^)] (Parsons *et al.*, 2013 ▸)	Flack *x* determined using 2891 quotients [(*I* ^+^)−(*I* ^−^)]/[(*I* ^+^)+(*I* ^−^)] (Parsons *et al.*, 2013 ▸)
Absolute structure parameter	−0.02 (3)	0.03 (4)	0.17 (13)	0.022 (14)

Computer programs: *APEX2* and *SAINT* (Bruker, 2012 ▸), *SORTAV* (Blessing, 1995 ▸), *SHELXS97* (Sheldrick, 2008 ▸), *SHELXL2018/3* (Sheldrick, 2015 ▸), and *ORTEP-3 for Windows* and *WinGX* publication routines (Farrugia, 2012 ▸).

## supplementary crystallographic information

### (S)-2-Butyl N-(4-nitrophenyl)thiocarbamate (I) Crystal data

**Table:**

C_11_H_14_N_2_O_3_S	*F*(000) = 536
*M**_r_* = 254.3	*D*_x_ = 1.339 Mg m^−^^3^
Monoclinic, *P*2_1_	Mo *K*α radiation, λ = 0.71073 Å
Hall symbol: P 2yb	Cell parameters from 8016 reflections
*a* = 16.052 (2) Å	θ = 2.5–32.3°
*b* = 4.7635 (6) Å	µ = 0.26 mm^−^^1^
*c* = 16.853 (2) Å	*T* = 100 K
β = 101.702 (8)°	Prism, yellow
*V* = 1261.9 (3) Å^3^	0.6 × 0.12 × 0.06 mm
*Z* = 4	

### (S)-2-Butyl N-(4-nitrophenyl)thiocarbamate (I) Data collection

**Table:**

Bruker APEXII diffractometer	9553 independent reflections
Radiation source: sealed x-ray tube	7648 reflections with *I* > 2σ(*I*)
Graphite monochromator	*R*_int_ = 0.047
φ or ω oscillation scans	θ_max_ = 33.3°, θ_min_ = 1.6°
Absorption correction: numerical (SADABS; Krause *et al.*, 2015)	*h* = −24→24
*T*_min_ = 0.959, *T*_max_ = 1	*k* = −7→7
37641 measured reflections	*l* = −25→25

### (S)-2-Butyl N-(4-nitrophenyl)thiocarbamate (I) Refinement

**Table:**

Refinement on *F*^2^	Secondary atom site location: difference Fourier map
Least-squares matrix: full	Hydrogen site location: mixed
*R*[*F*^2^ > 2σ(*F*^2^)] = 0.039	H atoms treated by a mixture of independent and constrained refinement
*wR*(*F*^2^) = 0.084	*w* = 1/[σ^2^(*F*_o_^2^) + (0.0311*P*)^2^ + 0.2393*P*] where *P* = (*F*_o_^2^ + 2*F*_c_^2^)/3
*S* = 1.01	(Δ/σ)_max_ < 0.001
9553 reflections	Δρ_max_ = 0.31 e Å^−^^3^
317 parameters	Δρ_min_ = −0.28 e Å^−^^3^
1 restraint	Absolute structure: Flack *x* determined using 2891 quotients [(*I*^+^)-(*I*^-^)]/[(*I*^+^)+(*I*^-^)] (Parsons *et al.*, 2013).
0 constraints	Absolute structure parameter: −0.02 (3)
Primary atom site location: structure-invariant direct methods	

### (S)-2-Butyl N-(4-nitrophenyl)thiocarbamate (I) Special details

**Table:**

Experimental. Crystals were mounted on a Cryoloop^TM^ (0.2–0.3mm, Hampton Research) with Paratone (R) oil. Between 7 to 12 data sets were collected to cover full Ewald spheres to a resolution of better than 0.75 Å. Crystals were held at 100 K with a Cryostream cooler, mounted to a Bruker APEXII single crystal X-ray diffractometer, Mo radiation (Bruker 2012), equipped with a fine-focus X-ray tube, Miracol X-ray optical collimator, and CCD detector. Crystal-to-detector distance was 40 mm and the exposure times were between 20 to 120 seconds per frame for all sets, pending on sample size. The scan widths were 0.5°. Crystal data, data collection, and structure refinement details are summarized in Table 5. The data were integrated and scaled using *SAINT*, *SADABS* within the APEX2 software package by Bruker (2012). Data work-up was done with *SAINT* (Bruker, 2012). Structures were solved with SHELXS (Sheldrick, 2008), and refined with SHELXL (Sheldrick 2015).
Geometry. All esds (except the esd in the dihedral angle between two l.s. planes) are estimated using the full covariance matrix. The cell esds are taken into account individually in the estimation of esds in distances, angles and torsion angles; correlations between esds in cell parameters are only used when they are defined by crystal symmetry. An approximate (isotropic) treatment of cell esds is used for estimating esds involving l.s. planes.

### (S)-2-Butyl N-(4-nitrophenyl)thiocarbamate (I) Fractional atomic coordinates and isotropic or equivalent isotropic displacement parameters (Å^2^)

**Table:**

	*x*	*y*	*z*	*U*_iso_*/*U*_eq_	
C1	0.15331 (12)	−0.1677 (4)	0.84542 (12)	0.0152 (4)	
C2	0.12449 (12)	−0.0015 (4)	0.77677 (12)	0.0174 (4)	
H2	0.151144	−0.016729	0.731506	0.021*	
C3	0.05799 (13)	0.1841 (4)	0.77393 (13)	0.0180 (4)	
H3	0.038254	0.295554	0.727155	0.022*	
C4	0.02066 (13)	0.2038 (4)	0.84101 (13)	0.0177 (4)	
C5	0.04839 (13)	0.0434 (5)	0.90953 (12)	0.0201 (4)	
H5	0.021711	0.061094	0.954713	0.024*	
C6	0.11491 (13)	−0.1429 (4)	0.91239 (13)	0.0198 (4)	
H6	0.134354	−0.253052	0.959486	0.024*	
C7	0.27251 (12)	−0.5046 (4)	0.89663 (12)	0.0160 (4)	
C8	0.39282 (13)	−0.4736 (6)	1.07282 (13)	0.0257 (4)	
H8A	0.423896	−0.579353	1.119423	0.031*	
H8B	0.382833	−0.281755	1.089701	0.031*	
H8C	0.426392	−0.468052	1.030338	0.031*	
C9	0.30844 (13)	−0.6152 (5)	1.04042 (12)	0.0194 (4)	
H9	0.318562	−0.810097	1.022493	0.023*	
C10	0.24760 (13)	−0.6239 (5)	1.09819 (13)	0.0223 (4)	
H10A	0.192737	−0.703946	1.069676	0.027*	
H10B	0.236767	−0.429797	1.114471	0.027*	
C11	0.28090 (15)	−0.7970 (5)	1.17392 (13)	0.0251 (5)	
H11A	0.235765	−0.819771	1.204789	0.03*	
H11B	0.32951	−0.700617	1.207476	0.03*	
H11C	0.298872	−0.981959	1.158234	0.03*	
C12	0.37889 (12)	−1.0550 (4)	0.66160 (12)	0.0145 (4)	
C13	0.41365 (12)	−1.2037 (4)	0.73232 (12)	0.0162 (4)	
H13	0.393993	−1.167115	0.780817	0.019*	
C14	0.47613 (12)	−1.4027 (4)	0.73227 (12)	0.0161 (4)	
H14	0.499536	−1.504145	0.780133	0.019*	
C15	0.50388 (11)	−1.4511 (4)	0.66101 (12)	0.0143 (3)	
C16	0.47042 (12)	−1.3078 (4)	0.59063 (12)	0.0171 (4)	
H16	0.490499	−1.345659	0.542426	0.021*	
C17	0.40740 (12)	−1.1085 (4)	0.59036 (12)	0.0168 (4)	
H17	0.383929	−1.009483	0.542073	0.02*	
C18	0.25910 (11)	−0.7200 (4)	0.61245 (11)	0.0144 (3)	
C19	0.23833 (14)	−0.7059 (6)	0.39684 (13)	0.0263 (4)	
H19A	0.199951	−0.630163	0.348897	0.032*	
H19B	0.250212	−0.903695	0.387626	0.032*	
H19C	0.291683	−0.599465	0.406889	0.032*	
C20	0.19702 (12)	−0.6818 (4)	0.46925 (11)	0.0164 (4)	
H20	0.186765	−0.479585	0.480041	0.02*	
C21	0.11459 (13)	−0.8446 (5)	0.46106 (14)	0.0230 (4)	
H21A	0.101425	−0.87198	0.515435	0.028*	
H21B	0.12168	−1.032074	0.437977	0.028*	
C22	0.04055 (13)	−0.6930 (6)	0.40690 (14)	0.0283 (5)	
H22A	−0.012029	−0.799175	0.405447	0.034*	
H22B	0.051502	−0.677764	0.351929	0.034*	
H22C	0.034616	−0.504726	0.428525	0.034*	
N1	0.21907 (11)	−0.3574 (4)	0.83957 (11)	0.0166 (3)	
H1N	0.2270 (15)	−0.384 (5)	0.7950 (15)	0.02*	
N2	−0.04965 (11)	0.3997 (4)	0.83882 (11)	0.0228 (4)	
N3	0.31755 (10)	−0.8517 (4)	0.66917 (10)	0.0151 (3)	
H3N	0.3146 (15)	−0.810 (5)	0.7178 (15)	0.018*	
N4	0.56963 (10)	−1.6622 (3)	0.66027 (10)	0.0173 (3)	
O1	−0.07477 (11)	0.5356 (4)	0.77657 (10)	0.0371 (4)	
O2	−0.08002 (10)	0.4226 (4)	0.89972 (10)	0.0296 (4)	
O3	0.26222 (9)	−0.4523 (3)	0.97083 (8)	0.0185 (3)	
O4	0.59552 (10)	−1.7972 (3)	0.72206 (9)	0.0257 (4)	
O5	0.59525 (9)	−1.6972 (4)	0.59691 (9)	0.0246 (3)	
O6	0.25984 (9)	−0.8002 (3)	0.53765 (8)	0.0168 (3)	
S1	0.34368 (3)	−0.72622 (11)	0.87190 (3)	0.01983 (11)	
S2	0.19349 (3)	−0.48150 (11)	0.63883 (3)	0.01827 (10)	

### (S)-2-Butyl N-(4-nitrophenyl)thiocarbamate (I) Atomic displacement parameters (Å^2^)

**Table:**

	*U*^11^	*U*^22^	*U*^33^	*U*^12^	*U*^13^	*U*^23^
C1	0.0164 (8)	0.0135 (8)	0.0158 (9)	−0.0006 (6)	0.0037 (7)	−0.0010 (7)
C2	0.0206 (9)	0.0176 (8)	0.0146 (9)	0.0004 (8)	0.0051 (7)	−0.0011 (8)
C3	0.0213 (9)	0.0176 (9)	0.0149 (9)	0.0007 (7)	0.0031 (7)	0.0008 (7)
C4	0.0175 (9)	0.0178 (9)	0.0177 (10)	0.0016 (7)	0.0033 (7)	−0.0020 (7)
C5	0.0214 (9)	0.0240 (10)	0.0162 (9)	0.0038 (8)	0.0065 (7)	0.0018 (8)
C6	0.0212 (10)	0.0227 (9)	0.0163 (10)	0.0034 (8)	0.0056 (8)	0.0025 (8)
C7	0.0182 (8)	0.0147 (8)	0.0156 (9)	−0.0001 (7)	0.0044 (7)	−0.0013 (7)
C8	0.0221 (10)	0.0343 (11)	0.0200 (10)	−0.0017 (10)	0.0025 (8)	0.0020 (10)
C9	0.0218 (10)	0.0208 (9)	0.0149 (9)	0.0033 (8)	0.0018 (7)	0.0019 (8)
C10	0.0243 (10)	0.0236 (10)	0.0194 (10)	−0.0026 (8)	0.0055 (8)	−0.0013 (8)
C11	0.0336 (12)	0.0241 (10)	0.0170 (10)	−0.0061 (9)	0.0039 (9)	−0.0004 (8)
C12	0.0130 (8)	0.0148 (8)	0.0152 (9)	−0.0002 (6)	0.0019 (7)	−0.0013 (7)
C13	0.0188 (8)	0.0174 (8)	0.0126 (9)	0.0019 (7)	0.0038 (7)	−0.0005 (8)
C14	0.0179 (9)	0.0173 (8)	0.0129 (9)	0.0024 (7)	0.0026 (7)	0.0005 (7)
C15	0.0129 (8)	0.0134 (8)	0.0166 (9)	0.0022 (6)	0.0028 (6)	0.0003 (7)
C16	0.0171 (9)	0.0206 (9)	0.0148 (9)	0.0018 (7)	0.0059 (7)	−0.0004 (7)
C17	0.0187 (9)	0.0185 (8)	0.0133 (9)	0.0038 (7)	0.0037 (7)	0.0017 (7)
C18	0.0129 (8)	0.0150 (7)	0.0151 (9)	−0.0006 (7)	0.0023 (6)	0.0009 (8)
C19	0.0225 (10)	0.0397 (12)	0.0163 (10)	−0.0003 (10)	0.0032 (8)	0.0027 (10)
C20	0.0166 (8)	0.0179 (9)	0.0134 (9)	0.0017 (7)	−0.0001 (7)	0.0022 (7)
C21	0.0200 (10)	0.0252 (10)	0.0221 (11)	−0.0021 (8)	0.0002 (8)	0.0034 (9)
C22	0.0172 (9)	0.0370 (13)	0.0285 (12)	0.0020 (9)	−0.0010 (8)	−0.0010 (11)
N1	0.0215 (8)	0.0175 (8)	0.0116 (8)	0.0042 (6)	0.0051 (6)	−0.0007 (6)
N2	0.0224 (9)	0.0266 (9)	0.0201 (9)	0.0061 (7)	0.0057 (7)	0.0007 (8)
N3	0.0165 (8)	0.0176 (7)	0.0110 (8)	0.0028 (6)	0.0021 (6)	−0.0008 (6)
N4	0.0175 (8)	0.0161 (7)	0.0178 (8)	0.0023 (6)	0.0025 (6)	−0.0002 (6)
O1	0.0385 (9)	0.0461 (10)	0.0284 (9)	0.0228 (9)	0.0113 (7)	0.0145 (9)
O2	0.0305 (9)	0.0396 (9)	0.0209 (8)	0.0139 (7)	0.0104 (7)	−0.0004 (7)
O3	0.0234 (7)	0.0201 (7)	0.0118 (6)	0.0052 (6)	0.0030 (5)	−0.0001 (6)
O4	0.0313 (8)	0.0245 (8)	0.0205 (8)	0.0126 (6)	0.0032 (6)	0.0050 (6)
O5	0.0247 (7)	0.0294 (8)	0.0224 (8)	0.0089 (7)	0.0107 (6)	−0.0004 (7)
O6	0.0168 (6)	0.0209 (7)	0.0119 (6)	0.0046 (5)	0.0007 (5)	0.0001 (5)
S1	0.0218 (2)	0.0214 (2)	0.0165 (2)	0.0062 (2)	0.00440 (18)	−0.0007 (2)
S2	0.0180 (2)	0.0205 (2)	0.0167 (2)	0.00655 (19)	0.00423 (18)	0.0005 (2)

### (S)-2-Butyl N-(4-nitrophenyl)thiocarbamate (I) Geometric parameters (Å, º)

**Table:**

C1—C6	1.396 (3)	C13—C14	1.380 (3)
C1—C2	1.401 (3)	C13—H13	0.95
C1—N1	1.408 (2)	C14—C15	1.382 (3)
C2—C3	1.379 (3)	C14—H14	0.95
C2—H2	0.95	C15—C16	1.379 (3)
C3—C4	1.386 (3)	C15—N4	1.460 (2)
C3—H3	0.95	C16—C17	1.387 (3)
C4—C5	1.381 (3)	C16—H16	0.95
C4—N2	1.459 (3)	C17—H17	0.95
C5—C6	1.382 (3)	C18—O6	1.320 (2)
C5—H5	0.95	C18—N3	1.350 (2)
C6—H6	0.95	C18—S2	1.669 (2)
C7—O3	1.318 (2)	C19—C20	1.507 (3)
C7—N1	1.348 (2)	C19—H19A	0.98
C7—S1	1.669 (2)	C19—H19B	0.98
C8—C9	1.512 (3)	C19—H19C	0.98
C8—H8A	0.98	C20—O6	1.481 (2)
C8—H8B	0.98	C20—C21	1.516 (3)
C8—H8C	0.98	C20—H20	1
C9—O3	1.475 (2)	C21—C22	1.525 (3)
C9—C10	1.513 (3)	C21—H21A	0.99
C9—H9	1	C21—H21B	0.99
C10—C11	1.522 (3)	C22—H22A	0.98
C10—H10A	0.99	C22—H22B	0.98
C10—H10B	0.99	C22—H22C	0.98
C11—H11A	0.98	N1—H1N	0.80 (2)
C11—H11B	0.98	N2—O2	1.227 (2)
C11—H11C	0.98	N2—O1	1.229 (2)
C12—C17	1.392 (3)	N3—H3N	0.85 (2)
C12—C13	1.401 (3)	N4—O4	1.223 (2)
C12—N3	1.405 (2)	N4—O5	1.231 (2)
			
C6—C1—C2	119.58 (18)	C13—C14—H14	120.7
C6—C1—N1	124.81 (18)	C15—C14—H14	120.7
C2—C1—N1	115.58 (17)	C16—C15—C14	121.78 (18)
C3—C2—C1	120.96 (19)	C16—C15—N4	119.32 (17)
C3—C2—H2	119.5	C14—C15—N4	118.90 (17)
C1—C2—H2	119.5	C15—C16—C17	119.87 (19)
C2—C3—C4	118.36 (19)	C15—C16—H16	120.1
C2—C3—H3	120.8	C17—C16—H16	120.1
C4—C3—H3	120.8	C16—C17—C12	119.32 (18)
C5—C4—C3	121.68 (18)	C16—C17—H17	120.3
C5—C4—N2	119.43 (18)	C12—C17—H17	120.3
C3—C4—N2	118.89 (18)	O6—C18—N3	113.71 (17)
C4—C5—C6	120.01 (19)	O6—C18—S2	125.43 (14)
C4—C5—H5	120	N3—C18—S2	120.85 (15)
C6—C5—H5	120	C20—C19—H19A	109.5
C5—C6—C1	119.41 (19)	C20—C19—H19B	109.5
C5—C6—H6	120.3	H19A—C19—H19B	109.5
C1—C6—H6	120.3	C20—C19—H19C	109.5
O3—C7—N1	113.22 (17)	H19A—C19—H19C	109.5
O3—C7—S1	125.47 (15)	H19B—C19—H19C	109.5
N1—C7—S1	121.30 (15)	O6—C20—C19	105.04 (15)
C9—C8—H8A	109.5	O6—C20—C21	108.67 (15)
C9—C8—H8B	109.5	C19—C20—C21	114.00 (18)
H8A—C8—H8B	109.5	O6—C20—H20	109.7
C9—C8—H8C	109.5	C19—C20—H20	109.7
H8A—C8—H8C	109.5	C21—C20—H20	109.7
H8B—C8—H8C	109.5	C20—C21—C22	111.86 (18)
O3—C9—C8	108.78 (18)	C20—C21—H21A	109.2
O3—C9—C10	103.89 (16)	C22—C21—H21A	109.2
C8—C9—C10	115.33 (17)	C20—C21—H21B	109.2
O3—C9—H9	109.5	C22—C21—H21B	109.2
C8—C9—H9	109.5	H21A—C21—H21B	107.9
C10—C9—H9	109.5	C21—C22—H22A	109.5
C9—C10—C11	113.06 (18)	C21—C22—H22B	109.5
C9—C10—H10A	109	H22A—C22—H22B	109.5
C11—C10—H10A	109	C21—C22—H22C	109.5
C9—C10—H10B	109	H22A—C22—H22C	109.5
C11—C10—H10B	109	H22B—C22—H22C	109.5
H10A—C10—H10B	107.8	C7—N1—C1	131.41 (18)
C10—C11—H11A	109.5	C7—N1—H1N	112.9 (18)
C10—C11—H11B	109.5	C1—N1—H1N	115.7 (18)
H11A—C11—H11B	109.5	O2—N2—O1	123.36 (19)
C10—C11—H11C	109.5	O2—N2—C4	118.18 (18)
H11A—C11—H11C	109.5	O1—N2—C4	118.46 (18)
H11B—C11—H11C	109.5	C18—N3—C12	130.92 (18)
C17—C12—C13	119.81 (17)	C18—N3—H3N	114.0 (16)
C17—C12—N3	124.28 (18)	C12—N3—H3N	115.0 (16)
C13—C12—N3	115.89 (17)	O4—N4—O5	123.50 (17)
C14—C13—C12	120.70 (18)	O4—N4—C15	118.46 (17)
C14—C13—H13	119.6	O5—N4—C15	118.04 (16)
C12—C13—H13	119.6	C7—O3—C9	121.03 (16)
C13—C14—C15	118.51 (18)	C18—O6—C20	119.80 (15)

### (S)-2-Butyl N-(4-nitrophenyl)thiocarbamate (I) Hydrogen-bond geometry (Å, º)

**Table:**

*D*—H···*A*	*D*—H	H···*A*	*D*···*A*	*D*—H···*A*
N1—H1*N*···S2	0.80 (2)	2.62 (2)	3.3762 (19)	159 (2)
N3—H3*N*···S1	0.85 (2)	2.57 (2)	3.4095 (18)	166 (2)
C2—H2···S2	0.95	2.87	3.592 (2)	134
C13—H13···S1	0.95	2.81	3.611 (2)	142
C5—H5···O2^i^	0.95	2.53	3.203 (3)	128

Symmetry code: (i) −*x*, *y*−1/2, −*z*+2.

### (S)-2-Butyl N-(4-methoxyphenyl)thiocarbamate (II) Crystal data

**Table:**

C_12_H_17_NO_2_S	*F*(000) = 512
*M**_r_* = 239.32	*D*_x_ = 1.249 Mg m^−^^3^
Monoclinic, *P*2_1_	Mo *K*α radiation, λ = 0.71073 Å
Hall symbol: P 2yb	Cell parameters from 21109 reflections
*a* = 6.6973 (5) Å	θ = 1.9–30.8°
*b* = 21.2076 (17) Å	µ = 0.24 mm^−^^1^
*c* = 9.1899 (7) Å	*T* = 100 K
β = 102.868 (5)°	Needle, colorless
*V* = 1272.49 (17) Å^3^	0.6 × 0.48 × 0.2 mm
*Z* = 4	

### (S)-2-Butyl N-(4-methoxyphenyl)thiocarbamate (II) Data collection

**Table:**

Bruker APEXII diffractometer	7694 independent reflections
Radiation source: sealed x-ray tube	5666 reflections with *I* > 2σ(*I*)
Graphite monochromator	*R*_int_ = 0.048
φ or ω oscillation scans	θ_max_ = 30.8°, θ_min_ = 1.9°
Absorption correction: numerical (SADABS; Krause *et al.*, 2015)	*h* = −9→9
*T*_min_ = 0.657, *T*_max_ = 0.745	*k* = −30→30
21043 measured reflections	*l* = −13→13

### (S)-2-Butyl N-(4-methoxyphenyl)thiocarbamate (II) Refinement

**Table:**

Refinement on *F*^2^	Secondary atom site location: difference Fourier map
Least-squares matrix: full	Hydrogen site location: mixed
*R*[*F*^2^ > 2σ(*F*^2^)] = 0.051	H atoms treated by a mixture of independent and constrained refinement
*wR*(*F*^2^) = 0.113	*w* = 1/[σ^2^(*F*_o_^2^) + (0.037*P*)^2^ + 0.3946*P*] where *P* = (*F*_o_^2^ + 2*F*_c_^2^)/3
*S* = 1.01	(Δ/σ)_max_ = 0.001
7694 reflections	Δρ_max_ = 0.51 e Å^−^^3^
368 parameters	Δρ_min_ = −0.42 e Å^−^^3^
293 restraints	Absolute structure: Flack *x* determined using 2891 quotients [(*I*^+^)-(*I*^-^)]/[(*I*^+^)+(*I*^-^)] (Parsons *et al.*, 2013).
0 constraints	Absolute structure parameter: 0.03 (4)
Primary atom site location: structure-invariant direct methods	

### (S)-2-Butyl N-(4-methoxyphenyl)thiocarbamate (II) Special details

**Table:**

Experimental. Crystals were mounted on a Cryoloop^TM^ (0.2–0.3mm, Hampton Research) with Paratone (R) oil. Between 7 to 12 data sets were collected to cover full Ewald spheres to a resolution of better than 0.75 Å. Crystals were held at 100 K with a Cryostream cooler, mounted to a Bruker APEXII single crystal X-ray diffractometer, Mo radiation (Bruker 2012), equipped with a fine-focus X-ray tube, Miracol X-ray optical collimator, and CCD detector. Crystal-to-detector distance was 40 mm and the exposure times were between 20 to 120 seconds per frame for all sets, pending on sample size. The scan widths were 0.5°. Crystal data, data collection, and structure refinement details are summarized in Table 5. The data were integrated and scaled using *SAINT*, *SADABS* within the APEX2 software package by Bruker (2012). Data work-up was done with *SAINT* (Bruker, 2012). Structures were solved with SHELXS (Sheldrick, 2008), and refined with SHELXL (Sheldrick 2015).
Geometry. All esds (except the esd in the dihedral angle between two l.s. planes) are estimated using the full covariance matrix. The cell esds are taken into account individually in the estimation of esds in distances, angles and torsion angles; correlations between esds in cell parameters are only used when they are defined by crystal symmetry. An approximate (isotropic) treatment of cell esds is used for estimating esds involving l.s. planes.

### (S)-2-Butyl N-(4-methoxyphenyl)thiocarbamate (II) Fractional atomic coordinates and isotropic or equivalent isotropic displacement parameters (Å^2^)

**Table:**

	*x*	*y*	*z*	*U*_iso_*/*U*_eq_	Occ. (<1)
C1	0.3234 (5)	0.72968 (17)	0.0597 (4)	0.0242 (8)	
C2	0.2356 (5)	0.72153 (15)	−0.0890 (4)	0.0245 (7)	
H2	0.094609	0.710973	−0.119493	0.029*	
C3	0.3544 (6)	0.72883 (15)	−0.1957 (4)	0.0233 (7)	
H3	0.295113	0.722886	−0.298681	0.028*	
C4	0.5579 (5)	0.74471 (18)	−0.1494 (4)	0.0269 (7)	
C5	0.6459 (5)	0.7519 (2)	0.0015 (4)	0.0333 (8)	
H5	0.787173	0.761973	0.032826	0.04*	
C6	0.5286 (5)	0.7444 (2)	0.1060 (4)	0.0340 (8)	
H6	0.58849	0.749394	0.209183	0.041*	
S1	0.02973 (14)	0.74842 (5)	0.39257 (10)	0.0343 (2)	
C7	0.1630 (5)	0.76439 (17)	0.2634 (4)	0.0278 (8)	
O1	0.2409 (4)	0.81978 (12)	0.2408 (3)	0.0355 (6)	
C8	0.444 (2)	0.9025 (7)	0.378 (3)	0.041 (2)	0.444 (4)
H8A	0.523678	0.868668	0.436354	0.061*	0.444 (4)
H8B	0.445317	0.939758	0.441278	0.061*	0.444 (4)
H8C	0.504598	0.913242	0.293399	0.061*	0.444 (4)
C9	0.229 (2)	0.8810 (7)	0.321 (2)	0.034 (2)	0.444 (4)
H9A	0.165251	0.873017	0.408306	0.041*	0.444 (4)
C10	0.1038 (14)	0.9302 (4)	0.2216 (12)	0.0382 (19)	0.444 (4)
H10A	0.093609	0.968145	0.282145	0.046*	0.444 (4)
H10B	0.177813	0.942314	0.143912	0.046*	0.444 (4)
C11	−0.1086 (17)	0.9096 (6)	0.1463 (15)	0.057 (3)	0.444 (4)
H11A	−0.100617	0.874981	0.077258	0.086*	0.444 (4)
H11B	−0.182427	0.945155	0.090835	0.086*	0.444 (4)
H11C	−0.181371	0.895306	0.221737	0.086*	0.444 (4)
C8B	0.393 (3)	0.9101 (8)	0.374 (3)	0.041 (2)	0.354 (4)
H8D	0.368205	0.949443	0.422835	0.061*	0.354 (4)
H8E	0.426634	0.919652	0.277802	0.061*	0.354 (4)
H8F	0.508114	0.887578	0.437639	0.061*	0.354 (4)
C9B	0.204 (3)	0.8694 (9)	0.349 (2)	0.034 (3)	0.354 (4)
H9B	0.1939	0.848955	0.445096	0.041*	0.354 (4)
C10B	0.0088 (18)	0.9043 (5)	0.2850 (13)	0.035 (2)	0.354 (4)
H10C	−0.108465	0.875614	0.282587	0.042*	0.354 (4)
H10D	−0.004249	0.939951	0.351796	0.042*	0.354 (4)
C11B	−0.003 (2)	0.9293 (5)	0.1310 (13)	0.039 (3)	0.354 (4)
H11D	−0.131226	0.952741	0.097632	0.058*	0.354 (4)
H11E	0.001174	0.894153	0.062582	0.058*	0.354 (4)
H11F	0.113275	0.957498	0.131893	0.058*	0.354 (4)
C8C	0.366 (4)	0.9172 (10)	0.333 (4)	0.041 (2)	0.202 (4)
H8G	0.33353	0.955948	0.380465	0.061*	0.202 (4)
H8H	0.395751	0.927403	0.235543	0.061*	0.202 (4)
H8I	0.486429	0.897102	0.396154	0.061*	0.202 (4)
C9C	0.187 (4)	0.8727 (15)	0.311 (5)	0.035 (3)	0.202 (4)
H9C	0.154489	0.861448	0.408593	0.042*	0.202 (4)
C10C	−0.005 (3)	0.8952 (9)	0.201 (3)	0.036 (3)	0.202 (4)
H10E	0.026609	0.899442	0.10069	0.043*	0.202 (4)
H10F	−0.113085	0.862847	0.193378	0.043*	0.202 (4)
C11C	−0.083 (3)	0.9564 (8)	0.243 (3)	0.041 (4)	0.202 (4)
H11G	−0.197989	0.970331	0.163991	0.062*	0.202 (4)
H11H	0.026427	0.98791	0.257494	0.062*	0.202 (4)
H11I	−0.129624	0.951219	0.336395	0.062*	0.202 (4)
C12	0.6008 (6)	0.7513 (2)	−0.3988 (4)	0.0370 (9)	
H12A	0.555585	0.707954	−0.424741	0.055*	
H12B	0.703873	0.763344	−0.454402	0.055*	
H12C	0.483348	0.779874	−0.424448	0.055*	
C13	−0.2448 (5)	0.59142 (17)	0.4748 (4)	0.0265 (8)	
C14	−0.1540 (5)	0.59857 (18)	0.6239 (4)	0.0275 (8)	
H14	−0.011877	0.607781	0.652848	0.033*	
C15	−0.2673 (5)	0.59251 (17)	0.7318 (4)	0.0269 (8)	
H15	−0.204178	0.597542	0.834465	0.032*	
C16	−0.4754 (5)	0.57890 (15)	0.6877 (4)	0.0224 (7)	
C17	−0.5672 (5)	0.57305 (19)	0.5371 (4)	0.0272 (7)	
H17	−0.709852	0.564724	0.507514	0.033*	
C18	−0.4541 (5)	0.57916 (18)	0.4312 (4)	0.0265 (8)	
H18	−0.517786	0.575071	0.328341	0.032*	
C19	−0.0790 (5)	0.55447 (17)	0.2769 (4)	0.0264 (8)	
C20	0.1078 (9)	0.4170 (3)	0.3247 (7)	0.081 (2)	
H20A	0.217303	0.447506	0.323526	0.122*	
H20B	0.140773	0.376905	0.282401	0.122*	
H20C	0.094917	0.410283	0.427702	0.122*	
C21	−0.0908 (7)	0.44190 (19)	0.2334 (6)	0.0484 (12)	
H21	−0.073927	0.452727	0.130872	0.058*	
C22	−0.2657 (10)	0.3961 (2)	0.2227 (6)	0.0655 (17)	
H22A	−0.227219	0.355534	0.183068	0.079*	
H22B	−0.287127	0.38815	0.324243	0.079*	
C23	−0.4637 (10)	0.4183 (3)	0.1252 (6)	0.0695 (18)	
H23A	−0.506915	0.457378	0.166255	0.104*	
H23B	−0.569058	0.385928	0.121697	0.104*	
H23C	−0.444471	0.426172	0.024169	0.104*	
C24	−0.5110 (6)	0.5739 (2)	0.9395 (4)	0.0342 (8)	
H24A	−0.401411	0.5425	0.965042	0.051*	
H24B	−0.615316	0.565495	0.996758	0.051*	
H24C	−0.453949	0.616142	0.96361	0.051*	
N1	0.2009 (5)	0.72059 (15)	0.1684 (3)	0.0284 (7)	
H1N	0.157 (6)	0.684 (2)	0.170 (5)	0.034*	
N2	−0.1264 (5)	0.59976 (16)	0.3643 (3)	0.0286 (7)	
H2N	−0.084 (6)	0.637 (2)	0.354 (4)	0.034*	
O2	0.6872 (3)	0.75506 (13)	−0.2435 (3)	0.0330 (6)	
O3	−0.1487 (4)	0.49852 (11)	0.3056 (3)	0.0317 (6)	
O4	−0.6018 (4)	0.57030 (13)	0.7839 (3)	0.0295 (5)	
S2	0.05382 (13)	0.56943 (4)	0.14638 (10)	0.0289 (2)	

### (S)-2-Butyl N-(4-methoxyphenyl)thiocarbamate (II) Atomic displacement parameters (Å^2^)

**Table:**

	*U*^11^	*U*^22^	*U*^33^	*U*^12^	*U*^13^	*U*^23^
C1	0.0204 (18)	0.0272 (17)	0.0252 (19)	0.0030 (13)	0.0060 (15)	−0.0071 (13)
C2	0.0197 (17)	0.0241 (16)	0.0278 (18)	0.0014 (13)	0.0014 (14)	−0.0046 (13)
C3	0.0241 (18)	0.0236 (16)	0.0215 (17)	0.0011 (13)	0.0034 (14)	−0.0012 (13)
C4	0.0185 (16)	0.0309 (17)	0.0314 (18)	0.0028 (14)	0.0058 (15)	−0.0042 (16)
C5	0.0150 (16)	0.045 (2)	0.037 (2)	0.0016 (16)	−0.0006 (15)	−0.0153 (19)
C6	0.0239 (18)	0.049 (2)	0.0251 (17)	0.0073 (18)	−0.0022 (15)	−0.0143 (18)
S1	0.0292 (5)	0.0490 (5)	0.0236 (4)	0.0062 (4)	0.0037 (4)	−0.0097 (4)
C7	0.0172 (16)	0.039 (2)	0.0231 (17)	0.0055 (14)	−0.0038 (14)	−0.0080 (14)
O1	0.0320 (14)	0.0382 (14)	0.0362 (15)	0.0014 (11)	0.0073 (12)	−0.0150 (12)
C8	0.046 (6)	0.027 (4)	0.052 (4)	−0.002 (4)	0.018 (5)	0.001 (3)
C9	0.040 (5)	0.036 (5)	0.030 (5)	0.001 (4)	0.016 (4)	−0.009 (4)
C10	0.042 (4)	0.031 (4)	0.043 (4)	0.001 (3)	0.015 (4)	−0.005 (3)
C11	0.040 (6)	0.052 (6)	0.076 (7)	0.000 (5)	0.006 (6)	0.012 (6)
C8B	0.046 (6)	0.027 (4)	0.052 (4)	−0.002 (4)	0.018 (5)	0.001 (3)
C9B	0.038 (5)	0.030 (5)	0.036 (6)	0.005 (4)	0.009 (5)	−0.011 (4)
C10B	0.042 (5)	0.029 (4)	0.036 (5)	0.002 (4)	0.013 (4)	−0.005 (4)
C11B	0.045 (7)	0.028 (5)	0.042 (6)	−0.001 (5)	0.009 (6)	0.008 (5)
C8C	0.046 (6)	0.027 (4)	0.052 (4)	−0.002 (4)	0.018 (5)	0.001 (3)
C9C	0.040 (6)	0.032 (5)	0.036 (6)	−0.001 (5)	0.015 (5)	−0.006 (5)
C10C	0.041 (6)	0.032 (6)	0.037 (6)	0.004 (5)	0.015 (6)	−0.003 (5)
C11C	0.046 (9)	0.024 (7)	0.057 (9)	−0.002 (7)	0.019 (8)	−0.004 (7)
C12	0.032 (2)	0.042 (2)	0.039 (2)	0.0034 (19)	0.0139 (18)	−0.0048 (19)
C13	0.0198 (18)	0.038 (2)	0.0214 (17)	0.0015 (14)	0.0032 (15)	−0.0062 (14)
C14	0.0148 (16)	0.044 (2)	0.0235 (18)	−0.0006 (15)	0.0032 (14)	−0.0062 (15)
C15	0.0209 (18)	0.0382 (19)	0.0199 (17)	0.0017 (14)	0.0005 (14)	−0.0058 (14)
C16	0.0208 (16)	0.0229 (17)	0.0259 (16)	0.0030 (13)	0.0105 (14)	−0.0036 (14)
C17	0.0145 (15)	0.0330 (17)	0.0331 (18)	0.0001 (15)	0.0034 (14)	−0.0046 (17)
C18	0.0202 (17)	0.036 (2)	0.0225 (16)	−0.0008 (14)	0.0025 (14)	−0.0049 (15)
C19	0.0166 (16)	0.043 (2)	0.0185 (16)	0.0067 (14)	0.0013 (13)	−0.0011 (14)
C20	0.080 (4)	0.071 (4)	0.109 (5)	0.046 (3)	0.058 (4)	0.041 (4)
C21	0.070 (3)	0.031 (2)	0.057 (3)	0.013 (2)	0.041 (3)	0.0039 (19)
C22	0.121 (5)	0.023 (2)	0.070 (3)	−0.001 (2)	0.060 (4)	0.002 (2)
C23	0.100 (5)	0.047 (3)	0.073 (4)	−0.036 (3)	0.044 (4)	−0.017 (3)
C24	0.036 (2)	0.042 (2)	0.0289 (18)	0.0073 (19)	0.0168 (17)	0.0025 (18)
N1	0.0259 (17)	0.0364 (17)	0.0223 (15)	0.0022 (13)	0.0041 (13)	−0.0085 (13)
N2	0.0248 (16)	0.0391 (17)	0.0242 (16)	−0.0037 (13)	0.0100 (14)	−0.0074 (13)
O2	0.0199 (12)	0.0426 (15)	0.0380 (14)	−0.0018 (11)	0.0099 (11)	−0.0088 (13)
O3	0.0351 (15)	0.0343 (14)	0.0300 (14)	0.0062 (11)	0.0163 (12)	0.0007 (11)
O4	0.0233 (12)	0.0393 (13)	0.0291 (12)	0.0005 (12)	0.0128 (10)	−0.0018 (12)
S2	0.0233 (4)	0.0415 (5)	0.0231 (4)	−0.0021 (4)	0.0079 (3)	−0.0047 (4)

### (S)-2-Butyl N-(4-methoxyphenyl)thiocarbamate (II) Geometric parameters (Å, º)

**Table:**

C1—C2	1.373 (5)	C9C—C10C	1.52 (2)
C1—C6	1.380 (5)	C9C—H9C	1
C1—N1	1.440 (4)	C10C—C11C	1.486 (18)
C2—C3	1.402 (5)	C10C—H10E	0.99
C2—H2	0.95	C10C—H10F	0.99
C3—C4	1.376 (5)	C11C—H11G	0.98
C3—H3	0.95	C11C—H11H	0.98
C4—O2	1.371 (4)	C11C—H11I	0.98
C4—C5	1.390 (5)	C12—O2	1.417 (4)
C5—C6	1.378 (5)	C12—H12A	0.98
C5—H5	0.95	C12—H12B	0.98
C6—H6	0.95	C12—H12C	0.98
S1—C7	1.671 (4)	C13—C14	1.378 (5)
C7—O1	1.320 (4)	C13—C18	1.394 (5)
C7—N1	1.337 (4)	C13—N2	1.432 (4)
O1—C9C	1.38 (4)	C14—C15	1.382 (5)
O1—C9B	1.50 (2)	C14—H14	0.95
O1—C9	1.51 (2)	C15—C16	1.392 (5)
C8—C9	1.490 (13)	C15—H15	0.95
C8—H8A	0.98	C16—O4	1.365 (4)
C8—H8B	0.98	C16—C17	1.389 (5)
C8—H8C	0.98	C17—C18	1.366 (5)
C9—C10	1.512 (14)	C17—H17	0.95
C9—H9A	1	C18—H18	0.95
C10—C11	1.501 (13)	C19—O3	1.323 (4)
C10—H10A	0.99	C19—N2	1.335 (4)
C10—H10B	0.99	C19—S2	1.674 (4)
C11—H11A	0.98	C20—C21	1.502 (7)
C11—H11B	0.98	C20—H20A	0.98
C11—H11C	0.98	C20—H20B	0.98
C8B—C9B	1.508 (16)	C20—H20C	0.98
C8B—H8D	0.98	C21—O3	1.466 (5)
C8B—H8E	0.98	C21—C22	1.508 (7)
C8B—H8F	0.98	C21—H21	1
C9B—C10B	1.504 (16)	C22—C23	1.501 (8)
C9B—H9B	1	C22—H22A	0.99
C10B—C11B	1.497 (13)	C22—H22B	0.99
C10B—H10C	0.99	C23—H23A	0.98
C10B—H10D	0.99	C23—H23B	0.98
C11B—H11D	0.98	C23—H23C	0.98
C11B—H11E	0.98	C24—O4	1.426 (4)
C11B—H11F	0.98	C24—H24A	0.98
C8C—C9C	1.51 (2)	C24—H24B	0.98
C8C—H8G	0.98	C24—H24C	0.98
C8C—H8H	0.98	N1—H1N	0.82 (4)
C8C—H8I	0.98	N2—H2N	0.86 (4)
			
C2—C1—C6	120.8 (3)	O1—C9C—H9C	110.8
C2—C1—N1	119.3 (3)	C8C—C9C—H9C	110.8
C6—C1—N1	119.9 (3)	C10C—C9C—H9C	110.8
C1—C2—C3	119.9 (3)	C11C—C10C—C9C	113 (2)
C1—C2—H2	120.1	C11C—C10C—H10E	109
C3—C2—H2	120.1	C9C—C10C—H10E	109
C4—C3—C2	119.2 (3)	C11C—C10C—H10F	109
C4—C3—H3	120.4	C9C—C10C—H10F	109
C2—C3—H3	120.4	H10E—C10C—H10F	107.8
O2—C4—C3	124.5 (3)	C10C—C11C—H11G	109.5
O2—C4—C5	115.2 (3)	C10C—C11C—H11H	109.5
C3—C4—C5	120.4 (3)	H11G—C11C—H11H	109.5
C6—C5—C4	120.1 (3)	C10C—C11C—H11I	109.5
C6—C5—H5	119.9	H11G—C11C—H11I	109.5
C4—C5—H5	119.9	H11H—C11C—H11I	109.5
C5—C6—C1	119.6 (3)	O2—C12—H12A	109.5
C5—C6—H6	120.2	O2—C12—H12B	109.5
C1—C6—H6	120.2	H12A—C12—H12B	109.5
O1—C7—N1	112.1 (3)	O2—C12—H12C	109.5
O1—C7—S1	125.7 (3)	H12A—C12—H12C	109.5
N1—C7—S1	122.1 (3)	H12B—C12—H12C	109.5
C7—O1—C9C	119.8 (15)	C14—C13—C18	120.0 (3)
C7—O1—C9B	112.9 (7)	C14—C13—N2	120.0 (3)
C7—O1—C9	128.6 (7)	C18—C13—N2	119.9 (3)
C9—C8—H8A	109.5	C13—C14—C15	120.8 (3)
C9—C8—H8B	109.5	C13—C14—H14	119.6
H8A—C8—H8B	109.5	C15—C14—H14	119.6
C9—C8—H8C	109.5	C14—C15—C16	118.9 (3)
H8A—C8—H8C	109.5	C14—C15—H15	120.5
H8B—C8—H8C	109.5	C16—C15—H15	120.5
C8—C9—O1	106.4 (12)	O4—C16—C17	115.6 (3)
C8—C9—C10	111.3 (12)	O4—C16—C15	124.3 (3)
O1—C9—C10	112.3 (12)	C17—C16—C15	120.1 (3)
C8—C9—H9A	108.9	C18—C17—C16	120.6 (3)
O1—C9—H9A	108.9	C18—C17—H17	119.7
C10—C9—H9A	108.9	C16—C17—H17	119.7
C11—C10—C9	114.8 (10)	C17—C18—C13	119.6 (3)
C11—C10—H10A	108.6	C17—C18—H18	120.2
C9—C10—H10A	108.6	C13—C18—H18	120.2
C11—C10—H10B	108.6	O3—C19—N2	112.5 (3)
C9—C10—H10B	108.6	O3—C19—S2	125.5 (3)
H10A—C10—H10B	107.5	N2—C19—S2	122.0 (3)
C10—C11—H11A	109.5	C21—C20—H20A	109.5
C10—C11—H11B	109.5	C21—C20—H20B	109.5
H11A—C11—H11B	109.5	H20A—C20—H20B	109.5
C10—C11—H11C	109.5	C21—C20—H20C	109.5
H11A—C11—H11C	109.5	H20A—C20—H20C	109.5
H11B—C11—H11C	109.5	H20B—C20—H20C	109.5
C9B—C8B—H8D	109.5	O3—C21—C20	109.0 (4)
C9B—C8B—H8E	109.5	O3—C21—C22	106.1 (3)
H8D—C8B—H8E	109.5	C20—C21—C22	112.8 (4)
C9B—C8B—H8F	109.5	O3—C21—H21	109.6
H8D—C8B—H8F	109.5	C20—C21—H21	109.6
H8E—C8B—H8F	109.5	C22—C21—H21	109.6
C10B—C9B—O1	110.0 (12)	C23—C22—C21	114.0 (4)
C10B—C9B—C8B	114.0 (14)	C23—C22—H22A	108.8
O1—C9B—C8B	104.2 (14)	C21—C22—H22A	108.8
C10B—C9B—H9B	109.5	C23—C22—H22B	108.8
O1—C9B—H9B	109.5	C21—C22—H22B	108.8
C8B—C9B—H9B	109.5	H22A—C22—H22B	107.6
C11B—C10B—C9B	113.7 (11)	C22—C23—H23A	109.5
C11B—C10B—H10C	108.8	C22—C23—H23B	109.5
C9B—C10B—H10C	108.8	H23A—C23—H23B	109.5
C11B—C10B—H10D	108.8	C22—C23—H23C	109.5
C9B—C10B—H10D	108.8	H23A—C23—H23C	109.5
H10C—C10B—H10D	107.7	H23B—C23—H23C	109.5
C10B—C11B—H11D	109.5	O4—C24—H24A	109.5
C10B—C11B—H11E	109.5	O4—C24—H24B	109.5
H11D—C11B—H11E	109.5	H24A—C24—H24B	109.5
C10B—C11B—H11F	109.5	O4—C24—H24C	109.5
H11D—C11B—H11F	109.5	H24A—C24—H24C	109.5
H11E—C11B—H11F	109.5	H24B—C24—H24C	109.5
C9C—C8C—H8G	109.5	C7—N1—C1	125.5 (3)
C9C—C8C—H8H	109.5	C7—N1—H1N	121 (3)
H8G—C8C—H8H	109.5	C1—N1—H1N	113 (3)
C9C—C8C—H8I	109.5	C19—N2—C13	125.6 (3)
H8G—C8C—H8I	109.5	C19—N2—H2N	119 (3)
H8H—C8C—H8I	109.5	C13—N2—H2N	116 (3)
O1—C9C—C8C	107 (3)	C4—O2—C12	116.9 (3)
O1—C9C—C10C	102 (2)	C19—O3—C21	120.2 (3)
C8C—C9C—C10C	115 (2)	C16—O4—C24	117.1 (3)

### (S)-2-Butyl N-(4-methoxyphenyl)thiocarbamate (II) Hydrogen-bond geometry (Å, º)

**Table:**

*D*—H···*A*	*D*—H	H···*A*	*D*···*A*	*D*—H···*A*
N1—H1*N*···S2	0.82 (4)	2.53 (4)	3.347 (3)	171 (4)
N2—H2*N*···S1	0.86 (4)	2.47 (4)	3.314 (3)	165 (4)
C12—H12*B*···S1^i^	0.98	2.86	3.793 (4)	158
C24—H24*B*···S2^ii^	0.98	2.86	3.819 (4)	167
C18—H18···S2^iii^	0.95	2.98	3.730 (4)	136
C10*B*—H10*C*···S1	0.99	2.96	3.445 (11)	112

Symmetry codes: (i) *x*+1, *y*, *z*−1; (ii) *x*−1, *y*, *z*+1; (iii) *x*−1, *y*, *z*.

### (S)-2-Butyl N-(4-fluorophenyl)thiocarbamate (III) Crystal data

**Table:**

C_11_H_14_FNOS	*F*(000) = 480
*M**_r_* = 227.29	*D*_x_ = 1.314 Mg m^−^^3^
Monoclinic, *P*2_1_	Mo *K*α radiation, λ = 0.71073 Å
Hall symbol: P 2yb	Cell parameters from 1312 reflections
*a* = 6.9723 (13) Å	θ = 2.7–18.9°
*b* = 20.166 (3) Å	µ = 0.27 mm^−^^1^
*c* = 8.2818 (14) Å	*T* = 100 K
β = 99.403 (13)°	Prism, colourless
*V* = 1148.8 (3) Å^3^	0.5 × 0.1 × 0.05 mm
*Z* = 4	

### (S)-2-Butyl N-(4-fluorophenyl)thiocarbamate (III) Data collection

**Table:**

Bruker APEXII diffractometer	5263 independent reflections
Radiation source: sealed x-ray tube	2448 reflections with *I* > 2σ(*I*)
Graphite monochromator	*R*_int_ = 0.171
φ or ω oscillation scans	θ_max_ = 27.5°, θ_min_ = 2.0°
Absorption correction: multi-scan (SADABS; Krause *et al.*, 2015)	*h* = −9→9
*T*_min_ = 0.863, *T*_max_ = 1	*k* = −26→26
10466 measured reflections	*l* = −10→10

### (S)-2-Butyl N-(4-fluorophenyl)thiocarbamate (III) Refinement

**Table:**

Refinement on *F*^2^	Secondary atom site location: difference Fourier map
Least-squares matrix: full	Hydrogen site location: mixed
*R*[*F*^2^ > 2σ(*F*^2^)] = 0.068	H atoms treated by a mixture of independent and constrained refinement
*wR*(*F*^2^) = 0.143	*w* = 1/[σ^2^(*F*_o_^2^) + (0.0402*P*)^2^] where *P* = (*F*_o_^2^ + 2*F*_c_^2^)/3
*S* = 0.95	(Δ/σ)_max_ < 0.001
5263 reflections	Δρ_max_ = 0.45 e Å^−^^3^
257 parameters	Δρ_min_ = −0.52 e Å^−^^3^
2 restraints	Absolute structure: Flack *x* determined using 2891 quotients [(*I*^+^)-(*I*^-^)]/[(*I*^+^)+(*I*^-^)] (Parsons *et al.*, 2013).
0 constraints	Absolute structure parameter: 0.17 (13)
Primary atom site location: structure-invariant direct methods	

### (S)-2-Butyl N-(4-fluorophenyl)thiocarbamate (III) Special details

**Table:**

Experimental. Crystals were mounted on a Cryoloop^TM^ (0.2–0.3mm, Hampton Research) with Paratone (R) oil. Between 7 to 12 data sets were collected to cover full Ewald spheres to a resolution of better than 0.75 Å. Crystals were held at 100 K with a Cryostream cooler, mounted to a Bruker APEXII single crystal X-ray diffractometer, Mo radiation (Bruker 2012), equipped with a fine-focus X-ray tube, Miracol X-ray optical collimator, and CCD detector. Crystal-to-detector distance was 40 mm and the exposure times were between 20 to 120 seconds per frame for all sets, pending on sample size. The scan widths were 0.5°. Crystal data, data collection, and structure refinement details are summarized in Table 5. The data were integrated and scaled using *SAINT*, *SADABS* within the APEX2 software package by Bruker (2012). Data work-up was done with *SAINT* (Bruker, 2012). Structures were solved with SHELXS (Sheldrick, 2008), and refined with SHELXL (Sheldrick 2015).
Geometry. All esds (except the esd in the dihedral angle between two l.s. planes) are estimated using the full covariance matrix. The cell esds are taken into account individually in the estimation of esds in distances, angles and torsion angles; correlations between esds in cell parameters are only used when they are defined by crystal symmetry. An approximate (isotropic) treatment of cell esds is used for estimating esds involving l.s. planes.

### (S)-2-Butyl N-(4-fluorophenyl)thiocarbamate (III) Fractional atomic coordinates and isotropic or equivalent isotropic displacement parameters (Å^2^)

**Table:**

	*x*	*y*	*z*	*U*_iso_*/*U*_eq_	
C1	0.8966 (7)	0.6722 (3)	0.5978 (6)	0.021 (2)	
C2	1.0905 (8)	0.6846 (3)	0.6605 (5)	0.023 (2)	
H2	1.130829	0.688684	0.775314	0.028*	
C3	1.2256 (6)	0.6911 (3)	0.5552 (7)	0.024 (2)	
H3	1.35816	0.699572	0.598067	0.029*	
C4	1.1666 (7)	0.6852 (4)	0.3872 (6)	0.023 (2)	
C5	0.9726 (8)	0.6727 (3)	0.3245 (5)	0.022 (2)	
H5	0.932364	0.66869	0.209695	0.027*	
C6	0.8376 (6)	0.6663 (3)	0.4298 (7)	0.022 (2)	
H6	0.70503	0.657801	0.386939	0.027*	
C7	0.7286 (12)	0.6991 (4)	0.8282 (12)	0.019 (2)	
C8	1.0523 (12)	0.8141 (4)	1.0396 (12)	0.032 (3)	
H8A	1.116505	0.772014	1.073864	0.048*	
H8B	1.065696	0.84452	1.133121	0.048*	
H8C	1.112971	0.833823	0.952221	0.048*	
C9	0.8394 (12)	0.8018 (4)	0.9774 (11)	0.019 (2)	
H9	0.775524	0.782224	1.06603	0.023*	
C10	0.7329 (12)	0.8630 (4)	0.9100 (11)	0.025 (2)	
H10A	0.801429	0.881895	0.824864	0.03*	
H10B	0.740265	0.896099	0.999075	0.03*	
C11	0.5181 (11)	0.8534 (4)	0.8352 (12)	0.034 (3)	
H11A	0.50897	0.826147	0.736278	0.051*	
H11B	0.458106	0.896705	0.80698	0.051*	
H11C	0.450137	0.831193	0.914735	0.051*	
C12	0.1978 (7)	0.5306 (3)	0.9155 (6)	0.019 (2)	
C13	0.0041 (8)	0.5172 (3)	0.8542 (5)	0.023 (2)	
H13	−0.036579	0.511975	0.739728	0.027*	
C14	−0.1301 (6)	0.5113 (3)	0.9604 (7)	0.023 (2)	
H14	−0.262522	0.502042	0.918484	0.028*	
C15	−0.0706 (8)	0.5188 (3)	1.1279 (7)	0.023 (2)	
C16	0.1231 (9)	0.5323 (3)	1.1892 (5)	0.025 (2)	
H16	0.163773	0.537508	1.303733	0.029*	
C17	0.2573 (6)	0.5382 (3)	1.0831 (7)	0.021 (2)	
H17	0.38972	0.547441	1.124978	0.026*	
C18	0.3719 (11)	0.5013 (4)	0.6901 (11)	0.014 (2)	
C19	0.4944 (12)	0.3531 (4)	0.6560 (11)	0.028 (2)	
H19A	0.605381	0.383457	0.678678	0.042*	
H19B	0.5244	0.317553	0.583497	0.042*	
H19C	0.468179	0.333968	0.758962	0.042*	
C20	0.3174 (12)	0.3908 (4)	0.5742 (11)	0.023 (2)	
H20	0.344348	0.410563	0.469454	0.028*	
C21	0.1363 (12)	0.3495 (4)	0.5410 (11)	0.026 (2)	
H21A	0.112572	0.330173	0.645849	0.031*	
H21B	0.160071	0.312194	0.468958	0.031*	
C22	−0.0480 (12)	0.3853 (4)	0.4618 (12)	0.031 (3)	
H22A	−0.080503	0.419931	0.535926	0.047*	
H22B	−0.155415	0.353511	0.440115	0.047*	
H22C	−0.0265	0.40553	0.358697	0.047*	
N1	0.7567 (10)	0.6619 (4)	0.7012 (9)	0.0215 (19)	
N2	0.3377 (10)	0.5404 (3)	0.8108 (9)	0.0188 (18)	
O1	0.8371 (7)	0.7537 (3)	0.8418 (7)	0.0201 (14)	
O2	0.2729 (8)	0.4444 (3)	0.6850 (7)	0.0220 (15)	
F1	1.2980 (7)	0.6908 (2)	0.2885 (7)	0.0307 (14)	
F2	−0.2026 (7)	0.5147 (3)	1.2271 (7)	0.0310 (13)	
S1	0.5732 (3)	0.67758 (11)	0.9542 (3)	0.0232 (6)	
S2	0.5266 (3)	0.52266 (12)	0.5636 (3)	0.0237 (6)	
H1N	0.682 (11)	0.628 (3)	0.681 (10)	0.028*	
H2N	0.384 (10)	0.580 (3)	0.832 (10)	0.028*	

### (S)-2-Butyl N-(4-fluorophenyl)thiocarbamate (III) Atomic displacement parameters (Å^2^)

**Table:**

	*U*^11^	*U*^22^	*U*^33^	*U*^12^	*U*^13^	*U*^23^
C1	0.026 (5)	0.017 (5)	0.020 (5)	0.000 (4)	0.005 (4)	−0.003 (5)
C2	0.022 (5)	0.024 (5)	0.024 (5)	0.005 (4)	0.004 (4)	−0.006 (5)
C3	0.017 (5)	0.028 (6)	0.026 (6)	0.002 (4)	0.001 (4)	−0.002 (5)
C4	0.020 (5)	0.020 (5)	0.032 (6)	0.003 (4)	0.012 (5)	−0.001 (5)
C5	0.019 (5)	0.016 (5)	0.032 (6)	−0.002 (4)	0.005 (4)	0.005 (5)
C6	0.015 (5)	0.019 (5)	0.031 (6)	−0.003 (4)	0.001 (4)	−0.004 (5)
C7	0.014 (5)	0.018 (5)	0.024 (6)	−0.001 (4)	0.000 (4)	0.001 (4)
C8	0.021 (6)	0.031 (6)	0.039 (7)	−0.003 (4)	−0.007 (5)	0.001 (5)
C9	0.021 (5)	0.015 (5)	0.022 (5)	−0.004 (4)	0.004 (4)	−0.005 (4)
C10	0.027 (5)	0.019 (5)	0.028 (6)	0.005 (4)	0.003 (4)	−0.005 (4)
C11	0.014 (5)	0.038 (6)	0.046 (7)	0.011 (4)	−0.001 (5)	0.006 (5)
C12	0.015 (5)	0.017 (5)	0.027 (5)	−0.007 (4)	0.006 (4)	−0.004 (5)
C13	0.010 (5)	0.019 (5)	0.038 (6)	0.007 (4)	0.000 (4)	0.003 (5)
C14	0.022 (5)	0.020 (6)	0.027 (6)	0.001 (4)	0.000 (5)	0.000 (5)
C15	0.024 (6)	0.025 (6)	0.023 (6)	0.000 (5)	0.014 (5)	0.010 (5)
C16	0.034 (6)	0.018 (5)	0.020 (5)	0.003 (4)	−0.001 (5)	−0.010 (4)
C17	0.017 (5)	0.022 (5)	0.025 (5)	−0.001 (4)	0.000 (4)	−0.003 (4)
C18	0.014 (5)	0.019 (5)	0.010 (5)	0.002 (4)	0.002 (4)	0.002 (4)
C19	0.024 (6)	0.028 (6)	0.032 (6)	0.012 (4)	0.004 (5)	−0.003 (5)
C20	0.024 (5)	0.023 (5)	0.022 (6)	−0.004 (4)	0.006 (4)	−0.008 (4)
C21	0.026 (5)	0.025 (5)	0.028 (6)	−0.008 (4)	0.009 (4)	−0.005 (5)
C22	0.022 (6)	0.031 (6)	0.038 (7)	−0.001 (4)	−0.001 (5)	0.002 (5)
N1	0.018 (5)	0.023 (5)	0.026 (5)	−0.007 (3)	0.011 (4)	−0.006 (4)
N2	0.015 (4)	0.016 (4)	0.027 (5)	−0.003 (3)	0.010 (4)	−0.005 (4)
O1	0.017 (3)	0.017 (3)	0.026 (4)	−0.007 (2)	0.004 (3)	0.000 (3)
O2	0.030 (4)	0.013 (3)	0.023 (4)	0.000 (3)	0.003 (3)	−0.007 (3)
F1	0.026 (3)	0.039 (4)	0.031 (3)	−0.002 (3)	0.016 (2)	0.003 (3)
F2	0.028 (3)	0.035 (3)	0.033 (3)	−0.001 (3)	0.012 (2)	−0.002 (3)
S1	0.0187 (13)	0.0246 (13)	0.0264 (14)	−0.0067 (10)	0.0038 (10)	−0.0008 (12)
S2	0.0196 (13)	0.0255 (13)	0.0264 (15)	−0.0052 (10)	0.0046 (10)	−0.0018 (12)

### (S)-2-Butyl N-(4-fluorophenyl)thiocarbamate (III) Geometric parameters (Å, º)

**Table:**

C1—C2	1.39	C12—C17	1.39
C1—C6	1.39	C12—N2	1.421 (8)
C1—N1	1.414 (8)	C13—C14	1.39
C2—C3	1.39	C13—H13	0.95
C2—H2	0.95	C14—C15	1.39
C3—C4	1.39	C14—H14	0.95
C3—H3	0.95	C15—F2	1.333 (6)
C4—F1	1.329 (6)	C15—C16	1.39
C4—C5	1.39	C16—C17	1.39
C5—C6	1.39	C16—H16	0.95
C5—H5	0.95	C17—H17	0.95
C6—H6	0.95	C18—N2	1.325 (10)
C7—O1	1.329 (9)	C18—O2	1.335 (9)
C7—N1	1.332 (11)	C18—S2	1.678 (9)
C7—S1	1.680 (9)	C19—C20	1.512 (10)
C8—C9	1.510 (10)	C19—H19A	0.98
C8—H8A	0.98	C19—H19B	0.98
C8—H8B	0.98	C19—H19C	0.98
C8—H8C	0.98	C20—O2	1.483 (10)
C9—O1	1.483 (10)	C20—C21	1.501 (11)
C9—C10	1.500 (11)	C20—H20	1
C9—H9	1	C21—C22	1.526 (11)
C10—C11	1.536 (10)	C21—H21A	0.99
C10—H10A	0.99	C21—H21B	0.99
C10—H10B	0.99	C22—H22A	0.98
C11—H11A	0.98	C22—H22B	0.98
C11—H11B	0.98	C22—H22C	0.98
C11—H11C	0.98	N1—H1N	0.86 (5)
C12—C13	1.39	N2—H2N	0.86 (5)
			
C2—C1—C6	120	C14—C13—H13	120
C2—C1—N1	121.7 (5)	C12—C13—H13	120
C6—C1—N1	118.2 (5)	C13—C14—C15	120
C1—C2—C3	120	C13—C14—H14	120
C1—C2—H2	120	C15—C14—H14	120
C3—C2—H2	120	F2—C15—C16	121.0 (5)
C4—C3—C2	120	F2—C15—C14	119.0 (5)
C4—C3—H3	120	C16—C15—C14	120
C2—C3—H3	120	C15—C16—C17	120
F1—C4—C5	120.8 (5)	C15—C16—H16	120
F1—C4—C3	119.2 (5)	C17—C16—H16	120
C5—C4—C3	120	C16—C17—C12	120
C6—C5—C4	120	C16—C17—H17	120
C6—C5—H5	120	C12—C17—H17	120
C4—C5—H5	120	N2—C18—O2	112.3 (7)
C5—C6—C1	120	N2—C18—S2	122.1 (6)
C5—C6—H6	120	O2—C18—S2	125.6 (7)
C1—C6—H6	120	C20—C19—H19A	109.5
O1—C7—N1	112.2 (8)	C20—C19—H19B	109.5
O1—C7—S1	125.2 (7)	H19A—C19—H19B	109.5
N1—C7—S1	122.5 (7)	C20—C19—H19C	109.5
C9—C8—H8A	109.5	H19A—C19—H19C	109.5
C9—C8—H8B	109.5	H19B—C19—H19C	109.5
H8A—C8—H8B	109.5	O2—C20—C21	105.3 (7)
C9—C8—H8C	109.5	O2—C20—C19	109.1 (7)
H8A—C8—H8C	109.5	C21—C20—C19	113.8 (8)
H8B—C8—H8C	109.5	O2—C20—H20	109.5
O1—C9—C10	108.2 (7)	C21—C20—H20	109.5
O1—C9—C8	104.7 (7)	C19—C20—H20	109.5
C10—C9—C8	113.0 (7)	C20—C21—C22	116.1 (7)
O1—C9—H9	110.2	C20—C21—H21A	108.3
C10—C9—H9	110.2	C22—C21—H21A	108.3
C8—C9—H9	110.2	C20—C21—H21B	108.3
C9—C10—C11	115.9 (7)	C22—C21—H21B	108.3
C9—C10—H10A	108.3	H21A—C21—H21B	107.4
C11—C10—H10A	108.3	C21—C22—H22A	109.5
C9—C10—H10B	108.3	C21—C22—H22B	109.5
C11—C10—H10B	108.3	H22A—C22—H22B	109.5
H10A—C10—H10B	107.4	C21—C22—H22C	109.5
C10—C11—H11A	109.5	H22A—C22—H22C	109.5
C10—C11—H11B	109.5	H22B—C22—H22C	109.5
H11A—C11—H11B	109.5	C7—N1—C1	126.9 (7)
C10—C11—H11C	109.5	C7—N1—H1N	116 (6)
H11A—C11—H11C	109.5	C1—N1—H1N	117 (6)
H11B—C11—H11C	109.5	C18—N2—C12	127.2 (7)
C13—C12—C17	120	C18—N2—H2N	126 (6)
C13—C12—N2	121.8 (5)	C12—N2—H2N	106 (6)
C17—C12—N2	118.1 (5)	C7—O1—C9	122.8 (7)
C14—C13—C12	120	C18—O2—C20	119.1 (7)

### (S)-2-Butyl N-(4-fluorophenyl)thiocarbamate (III) Hydrogen-bond geometry (Å, º)

**Table:**

*D*—H···*A*	*D*—H	H···*A*	*D*···*A*	*D*—H···*A*
N1—H1*N*···S2	0.86 (5)	2.51 (5)	3.341 (8)	164 (8)
N2—H2*N*···S1	0.86 (5)	2.50 (5)	3.336 (7)	165 (7)
C8—H8*A*···F1^i^	0.98	2.59	3.494 (10)	154

Symmetry code: (i) *x*, *y*, *z*+1.

### (S)-2-Butyl N-(4-chlorophenyl)thiocarbamate (IV) Crystal data

**Table:**

C_11_H_14_ClNOS	*F*(000) = 512
*M**_r_* = 243.74	*D*_x_ = 1.337 Mg m^−^^3^
Monoclinic, *P*2_1_	Mo *K*α radiation, λ = 0.71073 Å
Hall symbol: P 2yb	Cell parameters from 9275 reflections
*a* = 15.4173 (15) Å	θ = 2.6–33.1°
*b* = 5.0170 (5) Å	µ = 0.46 mm^−^^1^
*c* = 16.2502 (15) Å	*T* = 100 K
β = 105.592 (5)°	Prsm, colourless
*V* = 1210.7 (2) Å^3^	0.6 × 0.12 × 0.11 mm
*Z* = 4	

### (S)-2-Butyl N-(4-chlorophenyl)thiocarbamate (IV) Data collection

**Table:**

Bruker APEXII diffractometer	9292 independent reflections
Radiation source: sealed x-ray tube	8469 reflections with *I* > 2σ(*I*)
Graphite monochromator	*R*_int_ = 0.029
φ or ω oscillation scans	θ_max_ = 33.3°, θ_min_ = 2.1°
Absorption correction: numerical (SADABS; Krause *et al.*, 2015)	*h* = −23→23
*T*_min_ = 0.954, *T*_max_ = 1	*k* = −7→7
46534 measured reflections	*l* = −24→25

### (S)-2-Butyl N-(4-chlorophenyl)thiocarbamate (IV) Refinement

**Table:**

Refinement on *F*^2^	Secondary atom site location: difference Fourier map
Least-squares matrix: full	Hydrogen site location: mixed
*R*[*F*^2^ > 2σ(*F*^2^)] = 0.027	H atoms treated by a mixture of independent and constrained refinement
*wR*(*F*^2^) = 0.066	*w* = 1/[σ^2^(*F*_o_^2^) + (0.0312*P*)^2^ + 0.1769*P*] where *P* = (*F*_o_^2^ + 2*F*_c_^2^)/3
*S* = 1.04	(Δ/σ)_max_ = 0.001
9292 reflections	Δρ_max_ = 0.36 e Å^−^^3^
281 parameters	Δρ_min_ = −0.22 e Å^−^^3^
1 restraint	Absolute structure: Flack *x* determined using 2891 quotients [(*I*^+^)-(*I*^-^)]/[(*I*^+^)+(*I*^-^)] (Parsons *et al.*, 2013).
0 constraints	Absolute structure parameter: 0.022 (14)
Primary atom site location: structure-invariant direct methods	

### (S)-2-Butyl N-(4-chlorophenyl)thiocarbamate (IV) Special details

**Table:**

Experimental. Crystals were mounted on a Cryoloop^TM^ (0.2–0.3mm, Hampton Research) with Paratone (R) oil. Between 7 to 12 data sets were collected to cover full Ewald spheres to a resolution of better than 0.75 Å. Crystals were held at 100 K with a Cryostream cooler, mounted to a Bruker APEXII single crystal X-ray diffractometer, Mo radiation (Bruker 2012), equipped with a fine-focus X-ray tube, Miracol X-ray optical collimator, and CCD detector. Crystal-to-detector distance was 40 mm and the exposure times were between 20 to 120 seconds per frame for all sets, pending on sample size. The scan widths were 0.5°. Crystal data, data collection, and structure refinement details are summarized in Table 5. The data were integrated and scaled using *SAINT*, *SADABS* within the APEX2 software package by Bruker (2012). Data work-up was done with *SAINT* (Bruker, 2012). Structures were solved with SHELXS (Sheldrick, 2008), and refined with SHELXL (Sheldrick 2015).
Geometry. All esds (except the esd in the dihedral angle between two l.s. planes) are estimated using the full covariance matrix. The cell esds are taken into account individually in the estimation of esds in distances, angles and torsion angles; correlations between esds in cell parameters are only used when they are defined by crystal symmetry. An approximate (isotropic) treatment of cell esds is used for estimating esds involving l.s. planes.

### (S)-2-Butyl N-(4-chlorophenyl)thiocarbamate (IV) Fractional atomic coordinates and isotropic or equivalent isotropic displacement parameters (Å^2^)

**Table:**

	*x*	*y*	*z*	*U*_iso_*/*U*_eq_	
C1	0.35185 (9)	0.0662 (3)	0.14953 (8)	0.0139 (2)	
C2	0.41945 (10)	0.0839 (4)	0.10745 (9)	0.0190 (3)	
H2A	0.467553	0.206748	0.126558	0.023*	
C3	0.41621 (10)	−0.0787 (4)	0.03751 (10)	0.0192 (3)	
H3	0.461955	−0.066932	0.008467	0.023*	
C4	0.34615 (10)	−0.2577 (3)	0.01033 (9)	0.0166 (3)	
C5	0.27962 (11)	−0.2816 (4)	0.05240 (10)	0.0205 (3)	
H5	0.232416	−0.407588	0.033739	0.025*	
C6	0.28265 (10)	−0.1197 (3)	0.12206 (10)	0.0187 (3)	
H6	0.23729	−0.135039	0.151438	0.022*	
C7	0.40912 (9)	0.3612 (3)	0.27658 (9)	0.0143 (3)	
C8	0.58731 (11)	0.2067 (4)	0.41659 (10)	0.0234 (3)	
H8A	0.533102	0.191469	0.436836	0.035*	
H8B	0.63713	0.274749	0.46281	0.035*	
H8C	0.60323	0.030984	0.398713	0.035*	
C9	0.56953 (10)	0.3964 (3)	0.34164 (9)	0.0152 (3)	
H9	0.555206	0.577305	0.3603	0.018*	
C10	0.64685 (10)	0.4172 (3)	0.30082 (10)	0.0183 (3)	
H10A	0.658195	0.239034	0.279654	0.022*	
H10B	0.701819	0.471755	0.344903	0.022*	
C11	0.62976 (12)	0.6144 (4)	0.22712 (11)	0.0246 (3)	
H11A	0.578675	0.553323	0.180904	0.037*	
H11B	0.68351	0.627046	0.206055	0.037*	
H11C	0.616089	0.790011	0.246922	0.037*	
C12	0.15284 (10)	0.8652 (3)	0.36586 (9)	0.0139 (3)	
C13	0.08213 (10)	0.8722 (3)	0.40427 (9)	0.0177 (3)	
H13	0.03196	0.756607	0.384976	0.021*	
C14	0.08536 (10)	1.0489 (4)	0.47084 (9)	0.0188 (3)	
H14	0.037264	1.054228	0.497095	0.023*	
C15	0.15837 (10)	1.2168 (3)	0.49896 (9)	0.0156 (3)	
C16	0.22863 (10)	1.2164 (3)	0.46032 (10)	0.0173 (3)	
H16	0.278113	1.334668	0.479234	0.021*	
C17	0.22527 (9)	1.0408 (3)	0.39386 (9)	0.0163 (3)	
H17	0.272809	1.03958	0.366869	0.02*	
C18	0.09692 (9)	0.5412 (3)	0.24559 (8)	0.0143 (2)	
C19	−0.11540 (12)	0.3508 (5)	0.25809 (12)	0.0303 (4)	
H19A	−0.07721	0.226643	0.298634	0.045*	
H19B	−0.170239	0.25891	0.226207	0.045*	
H19C	−0.131607	0.50161	0.289328	0.045*	
C20	−0.06464 (10)	0.4518 (4)	0.19644 (10)	0.0196 (3)	
H20	−0.043938	0.297952	0.16748	0.024*	
C21	−0.11786 (11)	0.6453 (4)	0.13017 (11)	0.0285 (4)	
H21A	−0.133859	0.802766	0.159794	0.034*	
H21B	−0.174639	0.558583	0.098314	0.034*	
C22	−0.06701 (14)	0.7390 (6)	0.06654 (13)	0.0423 (6)	
H22A	−0.011127	0.827881	0.097427	0.063*	
H22B	−0.104658	0.864221	0.02596	0.063*	
H22C	−0.052677	0.58506	0.035482	0.063*	
N1	0.34513 (8)	0.2393 (3)	0.21636 (8)	0.0161 (2)	
H1	0.2928 (13)	0.275 (5)	0.2178 (12)	0.019*	
N2	0.15996 (8)	0.6818 (3)	0.30193 (8)	0.0148 (2)	
H2	0.2126 (13)	0.652 (4)	0.3009 (12)	0.018*	
O1	0.49217 (6)	0.2959 (2)	0.27480 (6)	0.0150 (2)	
O2	0.01363 (7)	0.5970 (2)	0.24906 (7)	0.0169 (2)	
S1	0.38309 (2)	0.57287 (9)	0.34614 (2)	0.02056 (8)	
S2	0.12456 (2)	0.32279 (9)	0.17890 (2)	0.01850 (8)	
Cl1	0.34068 (3)	−0.45232 (9)	−0.07979 (2)	0.02263 (8)	
Cl2	0.16375 (3)	1.43009 (8)	0.58491 (2)	0.02094 (8)	

### (S)-2-Butyl N-(4-chlorophenyl)thiocarbamate (IV) Atomic displacement parameters (Å^2^)

**Table:**

	*U*^11^	*U*^22^	*U*^33^	*U*^12^	*U*^13^	*U*^23^
C1	0.0125 (5)	0.0154 (6)	0.0131 (5)	0.0004 (6)	0.0022 (4)	−0.0009 (6)
C2	0.0149 (6)	0.0245 (8)	0.0182 (6)	−0.0054 (6)	0.0053 (5)	−0.0064 (6)
C3	0.0157 (6)	0.0250 (8)	0.0178 (6)	−0.0024 (6)	0.0059 (5)	−0.0054 (6)
C4	0.0165 (6)	0.0171 (7)	0.0147 (6)	0.0016 (5)	0.0017 (5)	−0.0034 (5)
C5	0.0186 (7)	0.0201 (8)	0.0231 (7)	−0.0055 (6)	0.0063 (6)	−0.0069 (6)
C6	0.0166 (7)	0.0210 (8)	0.0202 (6)	−0.0041 (6)	0.0075 (5)	−0.0038 (6)
C7	0.0133 (6)	0.0161 (7)	0.0135 (6)	−0.0012 (5)	0.0037 (5)	−0.0010 (5)
C8	0.0242 (8)	0.0261 (8)	0.0168 (7)	−0.0033 (7)	0.0002 (6)	0.0043 (6)
C9	0.0127 (6)	0.0160 (7)	0.0147 (6)	−0.0015 (5)	−0.0001 (5)	−0.0016 (5)
C10	0.0143 (6)	0.0180 (7)	0.0216 (7)	−0.0024 (6)	0.0032 (5)	0.0014 (6)
C11	0.0266 (8)	0.0219 (9)	0.0266 (8)	−0.0034 (6)	0.0092 (6)	0.0035 (6)
C12	0.0137 (6)	0.0148 (7)	0.0124 (6)	0.0009 (5)	0.0023 (5)	−0.0007 (5)
C13	0.0151 (6)	0.0216 (8)	0.0176 (6)	−0.0042 (5)	0.0061 (5)	−0.0040 (6)
C14	0.0168 (6)	0.0228 (7)	0.0181 (6)	−0.0011 (6)	0.0070 (5)	−0.0046 (6)
C15	0.0175 (6)	0.0149 (6)	0.0135 (6)	0.0021 (5)	0.0024 (5)	−0.0015 (5)
C16	0.0152 (6)	0.0175 (7)	0.0185 (6)	−0.0023 (6)	0.0031 (5)	−0.0029 (6)
C17	0.0141 (6)	0.0180 (7)	0.0175 (6)	−0.0015 (6)	0.0053 (5)	−0.0018 (6)
C18	0.0134 (6)	0.0161 (6)	0.0129 (5)	−0.0006 (5)	0.0028 (4)	0.0004 (5)
C19	0.0210 (8)	0.0366 (11)	0.0354 (9)	−0.0074 (8)	0.0112 (7)	−0.0048 (9)
C20	0.0119 (6)	0.0239 (8)	0.0218 (7)	−0.0025 (6)	0.0023 (5)	−0.0080 (6)
C21	0.0180 (7)	0.0377 (11)	0.0254 (8)	0.0058 (7)	−0.0017 (6)	−0.0054 (7)
C22	0.0366 (11)	0.0611 (16)	0.0255 (9)	0.0153 (11)	0.0020 (8)	0.0125 (10)
N1	0.0107 (5)	0.0209 (6)	0.0167 (6)	−0.0008 (5)	0.0037 (4)	−0.0051 (5)
N2	0.0107 (5)	0.0181 (6)	0.0161 (5)	−0.0008 (5)	0.0044 (4)	−0.0038 (5)
O1	0.0113 (4)	0.0179 (5)	0.0146 (4)	−0.0005 (4)	0.0017 (3)	−0.0031 (4)
O2	0.0109 (4)	0.0210 (6)	0.0179 (5)	−0.0004 (4)	0.0025 (4)	−0.0059 (4)
S1	0.01572 (15)	0.0266 (2)	0.01970 (16)	−0.00029 (16)	0.00542 (12)	−0.00983 (16)
S2	0.01356 (15)	0.02352 (19)	0.01815 (16)	0.00004 (15)	0.00376 (12)	−0.00788 (15)
Cl1	0.02137 (17)	0.02587 (19)	0.01964 (16)	0.00053 (16)	0.00377 (13)	−0.00968 (16)
Cl2	0.02176 (17)	0.02132 (18)	0.01908 (16)	0.00135 (15)	0.00432 (13)	−0.00738 (14)

### (S)-2-Butyl N-(4-chlorophenyl)thiocarbamate (IV) Geometric parameters (Å, º)

**Table:**

C1—C2	1.3945 (19)	C12—C17	1.399 (2)
C1—C6	1.397 (2)	C12—N2	1.4138 (19)
C1—N1	1.4160 (19)	C13—C14	1.389 (2)
C2—C3	1.389 (2)	C13—H13	0.95
C2—H2A	0.95	C14—C15	1.382 (2)
C3—C4	1.383 (2)	C14—H14	0.95
C3—H3	0.95	C15—C16	1.390 (2)
C4—C5	1.382 (2)	C15—Cl2	1.7436 (16)
C4—Cl1	1.7432 (16)	C16—C17	1.384 (2)
C5—C6	1.384 (2)	C16—H16	0.95
C5—H5	0.95	C17—H17	0.95
C6—H6	0.95	C18—O2	1.3301 (17)
C7—O1	1.3296 (17)	C18—N2	1.3425 (18)
C7—N1	1.3364 (19)	C18—S2	1.6747 (16)
C7—S1	1.6763 (15)	C19—C20	1.515 (2)
C8—C9	1.512 (2)	C19—H19A	0.98
C8—H8A	0.98	C19—H19B	0.98
C8—H8B	0.98	C19—H19C	0.98
C8—H8C	0.98	C20—O2	1.4708 (18)
C9—O1	1.4698 (17)	C20—C21	1.517 (3)
C9—C10	1.516 (2)	C20—H20	1
C9—H9	1	C21—C22	1.530 (3)
C10—C11	1.521 (2)	C21—H21A	0.99
C10—H10A	0.99	C21—H21B	0.99
C10—H10B	0.99	C22—H22A	0.98
C11—H11A	0.98	C22—H22B	0.98
C11—H11B	0.98	C22—H22C	0.98
C11—H11C	0.98	N1—H1	0.83 (2)
C12—C13	1.395 (2)	N2—H2	0.829 (19)
			
C2—C1—C6	119.55 (14)	C14—C13—H13	120.1
C2—C1—N1	123.74 (14)	C12—C13—H13	120.1
C6—C1—N1	116.58 (12)	C15—C14—C13	120.14 (13)
C3—C2—C1	119.78 (15)	C15—C14—H14	119.9
C3—C2—H2A	120.1	C13—C14—H14	119.9
C1—C2—H2A	120.1	C14—C15—C16	120.93 (14)
C4—C3—C2	119.74 (14)	C14—C15—Cl2	119.89 (12)
C4—C3—H3	120.1	C16—C15—Cl2	119.18 (12)
C2—C3—H3	120.1	C17—C16—C15	118.89 (14)
C5—C4—C3	121.20 (14)	C17—C16—H16	120.6
C5—C4—Cl1	119.48 (12)	C15—C16—H16	120.6
C3—C4—Cl1	119.31 (12)	C16—C17—C12	120.93 (13)
C4—C5—C6	119.16 (15)	C16—C17—H17	119.5
C4—C5—H5	120.4	C12—C17—H17	119.5
C6—C5—H5	120.4	O2—C18—N2	112.99 (13)
C5—C6—C1	120.55 (14)	O2—C18—S2	125.57 (11)
C5—C6—H6	119.7	N2—C18—S2	121.44 (11)
C1—C6—H6	119.7	C20—C19—H19A	109.5
O1—C7—N1	113.38 (13)	C20—C19—H19B	109.5
O1—C7—S1	125.26 (11)	H19A—C19—H19B	109.5
N1—C7—S1	121.35 (11)	C20—C19—H19C	109.5
C9—C8—H8A	109.5	H19A—C19—H19C	109.5
C9—C8—H8B	109.5	H19B—C19—H19C	109.5
H8A—C8—H8B	109.5	O2—C20—C19	105.68 (13)
C9—C8—H8C	109.5	O2—C20—C21	107.36 (14)
H8A—C8—H8C	109.5	C19—C20—C21	113.99 (14)
H8B—C8—H8C	109.5	O2—C20—H20	109.9
O1—C9—C8	108.35 (12)	C19—C20—H20	109.9
O1—C9—C10	106.11 (11)	C21—C20—H20	109.9
C8—C9—C10	113.72 (13)	C20—C21—C22	113.51 (15)
O1—C9—H9	109.5	C20—C21—H21A	108.9
C8—C9—H9	109.5	C22—C21—H21A	108.9
C10—C9—H9	109.5	C20—C21—H21B	108.9
C9—C10—C11	113.53 (13)	C22—C21—H21B	108.9
C9—C10—H10A	108.9	H21A—C21—H21B	107.7
C11—C10—H10A	108.9	C21—C22—H22A	109.5
C9—C10—H10B	108.9	C21—C22—H22B	109.5
C11—C10—H10B	108.9	H22A—C22—H22B	109.5
H10A—C10—H10B	107.7	C21—C22—H22C	109.5
C10—C11—H11A	109.5	H22A—C22—H22C	109.5
C10—C11—H11B	109.5	H22B—C22—H22C	109.5
H11A—C11—H11B	109.5	C7—N1—C1	130.59 (13)
C10—C11—H11C	109.5	C7—N1—H1	114.2 (15)
H11A—C11—H11C	109.5	C1—N1—H1	115.2 (15)
H11B—C11—H11C	109.5	C18—N2—C12	131.16 (13)
C13—C12—C17	119.34 (13)	C18—N2—H2	115.1 (14)
C13—C12—N2	124.73 (14)	C12—N2—H2	113.7 (14)
C17—C12—N2	115.86 (13)	C7—O1—C9	119.61 (11)
C14—C13—C12	119.74 (14)	C18—O2—C20	121.44 (12)

### (S)-2-Butyl N-(4-chlorophenyl)thiocarbamate (IV) Hydrogen-bond geometry (Å, º)

**Table:**

*D*—H···*A*	*D*—H	H···*A*	*D*···*A*	*D*—H···*A*
N1—H1···S2	0.83 (2)	2.511 (19)	3.3163 (13)	163.0 (18)
N2—H2···S1	0.829 (19)	2.563 (19)	3.3645 (13)	162.8 (17)
C6—H6···S2	0.95	2.99	3.5961 (17)	123
C17—H17···S1	0.95	2.97	3.6122 (16)	127
C2—H2*A*···O1	0.95	2.38	2.8539 (18)	111
C13—H13···O2	0.95	2.29	2.8197 (19)	114
